# Evolution of metabolic capabilities and molecular features of diplonemids, kinetoplastids, and euglenids

**DOI:** 10.1186/s12915-020-0754-1

**Published:** 2020-03-02

**Authors:** Anzhelika Butenko, Fred R. Opperdoes, Olga Flegontova, Aleš Horák, Vladimír Hampl, Patrick Keeling, Ryan M. R. Gawryluk, Denis Tikhonenkov, Pavel Flegontov, Julius Lukeš

**Affiliations:** 1 Institute of Parasitology, Biology Centre, Czech Academy of Sciences, České Budějovice (Budweis), Czech Republic; 2 Faculty of Science, University of Ostrava, Ostrava, Czech Republic; 3 de Duve Institute, Université Catholique de Louvain, Brussels, Belgium; 4 Faculty of Science, University of South Bohemia, České Budějovice (Budweis), Czech Republic; 5 Faculty of Science, Charles University, Biocev, Vestec, Czech Republic; 6 Department of Botany, University of British Columbia, Vancouver, Canada; 7 Department of Biology, University of Victoria, Victoria, Canada; 8 Papanin Institute for Biology of Inland Waters, Russian Academy of Sciences, Borok, Russia; 9 Present address: Department of Genetics, Harvard Medical School, Boston, USA

**Keywords:** Metabolism, Comparative genomics, Evolution, Kinetoplastea, Diplonemea, Euglenida, Kinetochores, Trypanothione

## Abstract

**Background:**

The Euglenozoa are a protist group with an especially rich history of evolutionary diversity. They include diplonemids, representing arguably the most species-rich clade of marine planktonic eukaryotes; trypanosomatids, which are notorious parasites of medical and veterinary importance; and free-living euglenids. These different lifestyles, and particularly the transition from free-living to parasitic, likely require different metabolic capabilities. We carried out a comparative genomic analysis across euglenozoan diversity to see how changing repertoires of enzymes and structural features correspond to major changes in lifestyles.

**Results:**

We find a gradual loss of genes encoding enzymes in the evolution of kinetoplastids, rather than a sudden decrease in metabolic capabilities corresponding to the origin of parasitism, while diplonemids and euglenids maintain more metabolic versatility. Distinctive characteristics of molecular machines such as kinetochores and the pre-replication complex that were previously considered specific to parasitic kinetoplastids were also identified in their free-living relatives. Therefore, we argue that they represent an ancestral rather than a derived state, as thought until the present. We also found evidence of ancient redundancy in systems such as NADPH-dependent thiol-redox. Only the genus *Euglena* possesses the combination of trypanothione-, glutathione-, and thioredoxin-based systems supposedly present in the euglenozoan common ancestor, while other representatives of the phylum have lost one or two of these systems. Lastly, we identified convergent losses of specific metabolic capabilities between free-living kinetoplastids and ciliates. Although this observation requires further examination, it suggests that certain eukaryotic lineages are predisposed to such convergent losses of key enzymes or whole pathways.

**Conclusions:**

The loss of metabolic capabilities might not be associated with the switch to parasitic lifestyle in kinetoplastids, and the presence of a highly divergent (or unconventional) kinetochore machinery might not be restricted to this protist group. The data derived from the transcriptomes of free-living early branching prokinetoplastids suggests that the pre-replication complex of Trypanosomatidae is a highly divergent version of the conventional machinery. Our findings shed light on trends in the evolution of metabolism in protists in general and open multiple avenues for future research.

## Background

Phylum Euglenozoa (Discoba, formerly Excavata) [1–3] represents a morphologically and phylogenetically well-defined and robust clade that is united on the basis of several shared ultrastructural and molecular features, such as the presence of a flagellar pocket and paraflagellar rod, and splice leader (SL) *trans*-splicing [4, 5]. The phylum consists of four main subclades—Euglenida, Kinetoplastea, Diplonemea, and Symbiontida—that exhibit remarkably different lifestyles and modes of nutrition, including predation, osmotrophy, parasitism, and photoautotrophy [6, 7].

Each of the four groups is supported by molecular trees and distinct characters. Euglenids are morphologically and ecologically diverse free-living flagellates that possess a number of shared features, including a prominent protein-rich pellicle, which in some species provides the cell with the capability of metabolic (pulsating) movement, and one or two flagella. Euglenids also use paramylon (β-(1,3)-glucan polysaccharide) as their storage compound (in contrast, for example, to starch and glycogen as major storage polysaccharides in plants and animals respectively). They include bacteriovorous (e.g., *Petalomonas*), eukaryovorous (e.g., *Peranema*), osmotrophic (e.g., *Rhabdomonas*), and photosynthetic lineages (e.g., *Euglena*) [8]. The latter acquired a secondary plastid of green algal origin [9] and some interesting functional differences from other plastids [10]. Although several high-coverage transcriptomic datasets have become available recently [11–14], obtaining chromosome-level assemblies for these organisms is complicated by large sizes of their genomes [15]. Symbiontids are a poorly studied lineage of anaerobic/microaerophilic flagellates with mitochondrion-like organelles localized under the cell surface, and ectosymbiotic prokaryotes covering the outer cell surface [16]. To date, no transcriptome or genomic data are publicly available for this group.

Until recently, diplonemids were considered a small group of only a few genera known primarily for their unusual mitochondrial genome with genes encoded in pieces and spliced together post-transcriptionally [17]. However, several new diplonemid species have recently been established in culture and described [18, 19]. Moreover, environmental sequencing studies revealed diplonemids to be among the most species-rich and diverse planktonic lineages in global oceans [20, 21]. These typically deep-sea pelagic diplonemids (DSPD) are morphologically diverse heterotrophic flagellates with large and complex nuclear genomes [22, 23].

Finally, kinetoplastids are a widespread class of free-living phagotrophic or parasitic protists. The parasites are very well studied and include the genera *Trypanosoma* and *Leishmania*, which are important pathogens of vertebrates including human [24]. Kinetoplastids usually have their mitochondrial DNA organized in a unique, eponymous structure termed the kinetoplast (k) DNA, which is composed of a densely packed network of thousands of mutually interlocked circular DNA molecules [25–27]. Kinetoplastea are divided into two groups, the early-branching Prokinetoplastina, containing the fish parasite *Ichthyobodo* and *Perkinsela* symbiont of amoebae, and Metakinetoplastina, which includes the parasitic Trypanosomatida and three recently established orders of predominantly free-living bodonids: Eubodonida, Parabodonida, and Neobodonida [28–30].

Kinetoplastids are notable for doing things differently than opisthokonts and can serve as a good example of eukaryotic diversity. The list of their molecular oddities, in some cases nevertheless shared with other groups of organisms, is extensive and includes the near absence of introns, base J, ubiquitous *trans*-splicing, the absence of transcriptional regulation of gene expression, extensive editing of mitochondrial mRNAs, divergent mitochondrial protein translocators and ribosomes, pre-replication complex, non-canonical kinetochore complex protein composition, and many other biochemical peculiarities, like the localization of glycolysis to the peroxisome-derived glycosomes and the presence of trypanothione-based thiol-redox system [24, 31–43]. It is often unclear which of these features may have evolved due to parasitic lifestyle of kinetoplastids and which originated more deeply in the evolution of this group.

In spite of the vast cell biological and ecological diversity within and between these groups, phylum Euglenozoa is highly supported in phylogenomics and has long been accepted. Their incredible variety can be challenging to explain and problematic for research methods, but it also provides a valuable opportunity to understand the evolutionary bases of cellular and metabolic innovations in a microbial eukaryotic group that has considerable environmental and medical importance. Until recently, there were insufficient data to conduct any comprehensive comparative genomic analyses across Euglenozoa as a whole, but the number of species for which complete (or nearly complete) genomic and transcriptomic data are available has increased rapidly. We are for the first time in possession of both a well-supported phylogenetic tree and the taxonomic distribution of a set of highly unusual characters, which together allow for a comprehensive character evolution analysis to understand the gain/loss, redundancy, and timing of key cellular innovations in euglenozoans. The potential of such analyses to overturn long-held assumptions is clear from similar studies on other lineages [44, 45], or on specific euglenozoan characters, like the origins of parasitism in trypanosomatids and presumed “parasite-specific” features [46, 47].

Here, we have undertaken a phylum-wide comparative genomics survey to understand the origin and evolution of metabolic and cellular innovations within Euglenozoa. We have sequenced transcriptomes from three diplonemids (*Hemistasia phaeocysticola*, *Rhynchopus humris*, and *Sulcionema specki*), a bodonid (*Trypanoplasma borreli*), and two free-living members of the deep-branching kinetoplastid lineage, Prokinetoplastina (PhF-6 and PhM-4). Together with publicly available data, this allows for reconstructions across 18 taxa and represents the most comprehensive and phylogenetically broad survey of the phylum to date.

## Results and discussion

### Phylogenomic analysis

**Fig. 1:**
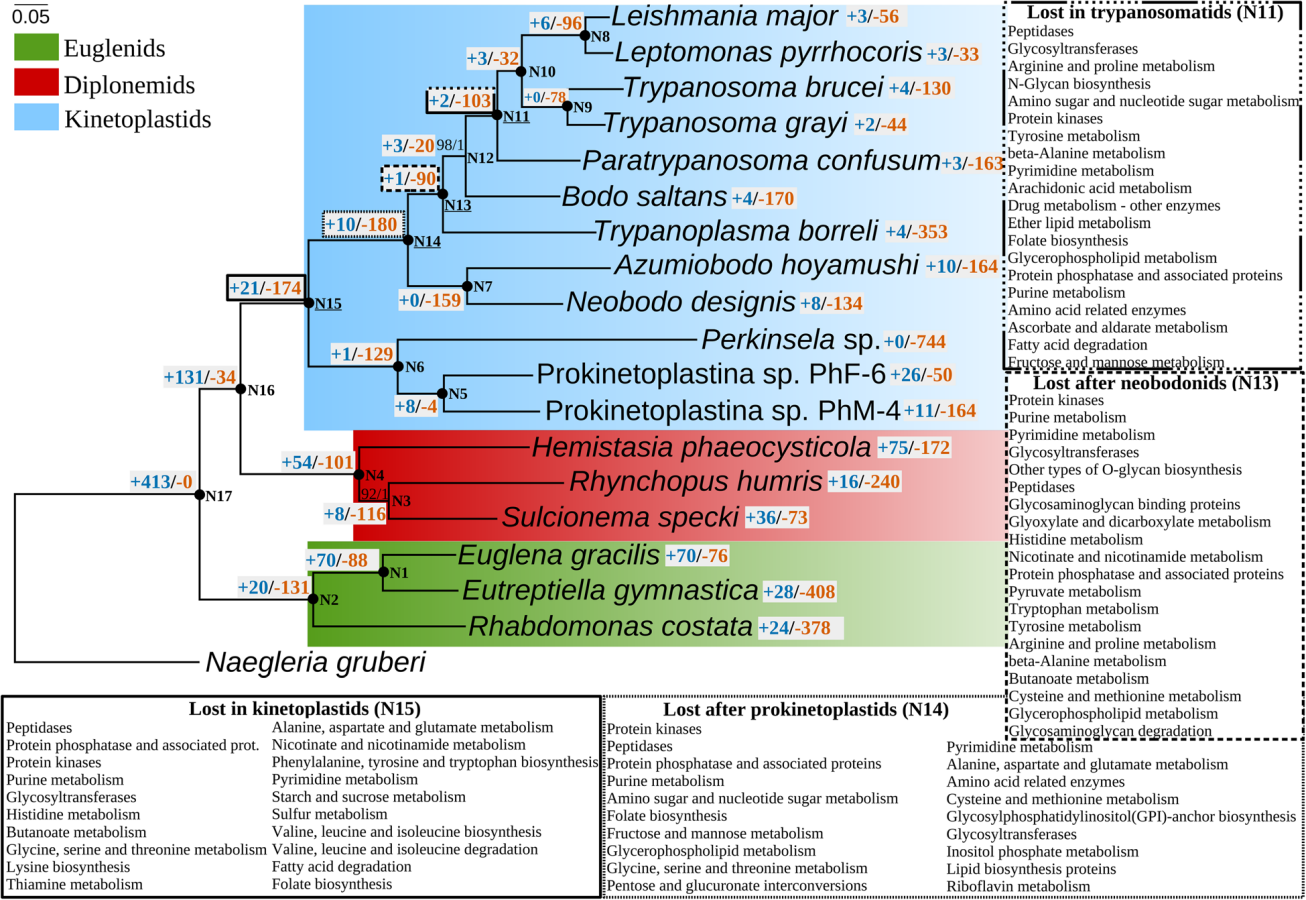
Gains and losses of metabolic functions in the evolution of Euglenozoa. The maximum-likelihood phylogenetic tree of Euglenozoa is based on a concatenated alignment of 20 conserved proteins (see the “Materials and methods” section). Nodes with 100% bootstrap support and posterior probability of 1.0 are marked with black circles. The tree was rooted with the sequences of a heterolobosean *Naegleria gruberi*. Kinetoplastids, diplonemids, and euglenids are shown on blue, red, and green background, respectively. The scale bar denotes the number of substitutions per site. Diplonemids and kinetoplastids represent sister clades with the maximal posterior probability and bootstrap support. Gains and losses of KEGG Orthology (KO) identifiers falling into the category “Metabolism” were mapped onto the phylogenetic tree using the Dollo parsimony algorithm implemented in the Count software v.10.4. Gains and losses are depicted on gray background in blue and vermillion, respectively. For each node of interest, KEGG Orthology categories with the highest number of lost KOs are shown

We inferred orthologous groups (OGs) for a set of proteins encoded in 19 protist genomes and transcriptomes, including three euglenid and three diplonemid transcriptomes, seven genomes and five transcriptomes of kinetoplastids, and the genome of *Naegleria gruberi* as an outgroup (Additional file 1: Table S1). For trypanosomatids, the most thoroughly studied kinetoplastid clade, we have included the sequences of *Paratrypanosoma confusum* and *Trypanosoma grayi*, which emerged as the slowest-evolving trypanosomatids in a recent study [48], along with genomes of model organisms *Trypanosoma brucei*, *Leishmania major*, and *Leptomonas pyrrhocoris* [49–51]*.* Sixty-three percent of the proteins in the initial dataset were clustered into 24,983 OGs, 52 of which contained one protein per species. In 20 of these 52 OGs, average protein identity was > 50% and the respective sequences were used for phylogenomic analysis (see the “Materials and methods” for details). The maximum-likelihood and Bayesian trees constructed based on these protein sequences displayed identical topologies, with almost all branches having maximal bootstrap supports and posterior probabilities (Fig. 1). In agreement with previous studies based on two proteins, our multi-gene phylogeny shows that diplonemids and kinetoplastids constitute sister clades, while euglenids are sister to both [31, 52].

### Overview of metabolic capabilities of euglenozoans

Completeness of the genome and transcriptome assemblies was assessed based on the presence of Benchmarking Universal Single-Copy Orthologs (BUSCOs) [53]. The results of this analysis indicate that the genomes and transcriptomes considered herein are representative, since a vast majority of them contained more than 70% of the universal eukaryotic genes (Additional file 1: Table S1). Three obvious exceptions are the apparently incomplete transcriptome of the euglenid *Eutreptiella gymnastica*, the genome of an obligate endosymbiont *Perkinsela* sp., which is the smallest kinetoplastid genome sequenced to date [30], and the transcriptome of the fish blood parasite *Trypanoplasma borreli* [54]. The first two assemblies lack approximately 37% of core eukaryotic genes, while the latter lacks ~ 31% (Additional file 1: Table S1). The most protein-rich transcriptomes are those of diplonemids (43,107 proteins on average), followed by euglenids (35,048 proteins; excluding the presumably incomplete transcriptome of *E. gymnastica*) and kinetoplastids, with the free-living and parasitic/endosymbiotic species encoding 23,894 and 10,970 proteins on average, respectively. The availability of genome sequences of sufficient quality for diplonemids and euglenids is a prerequisite for elucidating whether the pronounced differences in protein-coding capacities within Euglenozoa can be explained by substantial gene duplications in diplonemids and euglenids or by other mechanisms.

Functional annotation of metabolic proteins encoded in the genomes and transcriptomes of the analyzed species was performed by assigning KEGG Orthology (KO) identifiers using the BlastKOALA software v.2.1 [55]. The number of unique KEGG identifiers assigned to a full protein set of a given species was used as an estimator of the overall metabolic versatility of this species. According to our analyses taking into account only unique KEGG identifiers and thus mitigating the differences between genomic and transcriptomic data, the transcriptomes of diplonemids and euglenids encode a higher number of metabolic proteins than those of kinetoplastids, except for the free-living prokinetoplastids (Additional file 2: Fig. S1; unpaired *t* test *p* value = 0.0004, 95% confidence interval). The average number of unique KEGG identifiers belonging to the category “metabolism” is 1101 for diplonemids, 872 for euglenids, 974 for free-living prokinetoplastids, and 625 for other kinetoplastids. Parasitic and/or symbiotic organisms in our dataset possess fewer metabolic proteins than their free-living kin (Additional file 2: Fig. S1; unpaired *t* test *p* value = 0.0004, 95% confidence interval). However, the estimated numbers of unique metabolic proteins in the free-living *Bodo saltans* and *Neobodo designis* are at the lower limit for free-living flagellates and are close to the respective numbers in their parasitic relatives. Although the life cycle of *Rhynchopus humris*, a diplonemid isolated from lobsters and clams, is not known in detail yet, it includes free-living stages. Moreover, this flagellate can switch between the trophic and swimming stages in culture [18, 56] and therefore is in our analyses tentatively placed into the group of free-living protists.

In order to obtain a general picture of the similarities and differences in metabolic abilities between diplonemids, euglenids, and kinetoplastids, we have analyzed unique KO identifiers shared among them (Additional file 3: Fig. S2). Diplonemids and euglenids share 142 KO identifiers, which are absent in kinetoplastids. The number of unique metabolic KO identifiers restricted to kinetoplastids (122) is lower than the counts of diplonemid- or euglenid-specific identifiers (221 and 246, respectively), even though the number of kinetoplastid species in our dataset is much higher than that of other lineages. The annotations of enzymes specific to diplonemids, kinetoplastids, and those that are exclusively shared between diplonemids and euglenids, as well as among metabolically versatile diplonemids, euglenids, and free-living prokinetoplastids, were grouped according to the KEGG Orthology system. The results suggest that diplonemids and euglenids differ from kinetoplastids mainly in the repertoire of protein kinases and phosphatases, peptidases, glycosyltransferases, as well as enzymes of amino acid and nucleotide metabolism, and lipid biosynthesis (Additional file 3: Fig. S2). Diplonemids and euglenids exclusively share a greater number of KEGG categories (91) with still understudied free-living prokinetoplastids than with other kinetoplastids (60).

We have compared metabolic capabilities of free-living kinetoplastids with those of the representatives of other protist groups, trying to sample their diversity as widely as possible (Additional file 1: Table S2). Importantly, most free-living species listed in Additional file 1: Table S2 are bacteriovorous, similar to the free-living bodonids, except for the stramenopile *Thraustotheca clavata*, which obtains nutrients from decaying organic matter [57]. These results suggest that only the genomes of ciliates, which encode 770 metabolic enzymes on average, are similar in number of unique KO categories to the kinetoplastid genomes/transcriptomes (excluding free-living prokinetoplastids) containing 625 genes encoding metabolic proteins with unique KEGG identifiers (unpaired *t* test *p* value = 0.343, 95% confidence interval), while other free-living species analyzed appear to be metabolically more versatile (Additional file 4: Fig. S3; unpaired *t* test *p* value = 5.5E^−6^, 95% confidence interval). Kinetoplastids and ciliates share a number of losses of genes encoding peptidases, protein kinases and phosphatases, glycosyltransferases, enzymes acting in purine and pyrimidine metabolism, metabolism of amino acids and sugars, and vitamins and cofactors (Additional file 5: Fig. S4).

We mapped gains and losses of unique KEGG identifiers for metabolic proteins onto the phylogeny using the Count software v.10.4 (Fig. 1) [58]. The results indicate that metabolic genes were lost in all kinetoplastids (Node 15; Fig. 1) or within the kinetoplastid tree in a stepwise manner at the nodes after the Prokinetoplastina (N14) and Neobodonida (N13) split points, and in trypanosomatids (N11). Notably, the analysis by Dollo’s parsimony gives hints about pathways which underwent gains/losses in certain groups, while for the exact patterns of metabolic gains/losses in each particular species the reader is referred to the corresponding sections of the manuscript. Losses of metabolic genes mainly affect the metabolism of amino acids, nucleotides, cofactors, vitamins, and lipids and reflect major changes in the repertoire of protein kinases, phosphatases, peptidases, and glycosyltransferases. It was shown previously that *B. saltans*, the closest known free-living relative of the obligatory parasitic trypanosomatids, had already lost several complete metabolic pathways of amino acid, purine, folate, and ubiquinone biosynthesis and that these therefore did not represent “parasitic reduction” [59]. The much extended kinetoplastid dataset now shows that certain metabolic proteins and entire pathways were probably lost even earlier in the evolution of kinetoplastids and these losses are not obviously tied to a major change in lifestyle like the origin of parasitism.

**Fig. 2:**
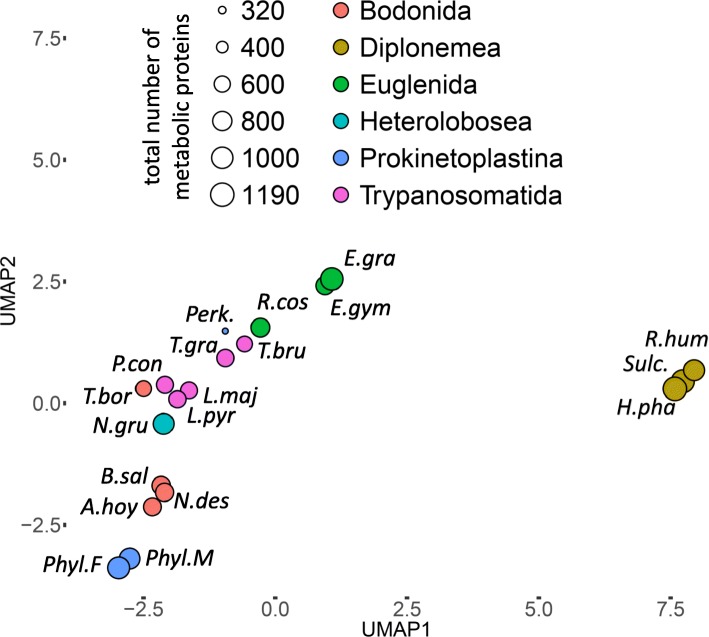
Species clustered according to their metabolic protein repertoires. A two-dimensional embedding of pairwise Hamming distances between presence/absence vectors of 2181 KEGG orthology (KO) identifiers was generated using the Uniform Manifold Approximation and Projection (UMAP) approach. The clades are color-coded according to the legend, and the total number of unique KO identifiers per species is coded by point size. The following species abbreviations are used: A.hoy, *Azumiobodo hoyamushi*; B.sal, *Bodo saltans*; E.gym, *Eutreptiella gymnastica*; E.gra, *Euglena gracilis*; H.pha, *Hemistasia phaeocysticola*; L.maj, *Leishmania major*; L.pyr, *Leptomonas pyrrhocoris*; N.des, *Neobodo designis*; N.gru, *Naegleria gruberi*; P.con, *Paratrypanosoma confusum*; Perk, *Perkinsela* sp.; Phyl. F, prokinetoplastid species PhF-6; Phyl. M, prokinetoplastid species PhM-4; R.cos, *Rhabdomonas costata*; R.hum, *Rhynchopus humris*; Sulc., *Sulcionema specki*; T.bor, *Trypanoplasma borreli*; T.bru, *Trypanosoma brucei*; T.gra, *Trypanosoma grayi*

We applied a Uniform Manifold Approximation and Projection (UMAP) approach [60] to see a general picture of species clustering according to their repertoires of metabolic proteins (unique KEGG identifiers). As described in the “Materials and methods” section, at first, we optimized UMAP settings and showed that the clustering is stable across analysis iterations run with different random seeds (Additional file 6: Fig. S5; Additional file 7: Fig. S6). A two-dimensional embedding of 2181-dimensional KEGG ID presence–absence vectors is shown in Fig. 2. The following six clusters are visible: (1) three diplonemids clustered tightly and lying far away from the other species; (2) the photosynthetic euglenids *Euglena gracilis* and *E. gymnastica*; (3) both free-living prokinetoplastids; (4) free-living bodonids *B. saltans* and *N. designis* and a parasitic neobodonid *Azumiobodo hoyamushi*; (5) a diverse cluster including a free-living heterolobosean *N. gruberi*, a specialized endoparasitic parabodonid *T*. *borreli*, and trypanosomatid parasites *P. confusum*, *L. major*, and *L*. *pyrrhocoris*; and (6) finally, a cluster composed of species having probably the most streamlined metabolism: parasitic trypanosomes, an obligatory endosymbiont *Perkinsela* sp., and a euglenid *Rhabdomonas costata*.

In order to check if the clustering recovered using UMAP reflects enzyme presence/absence patterns, we repeated an analysis of intersections, but this time species groups were defined not according to taxonomy, but according to the UMAP results (Additional file 8: Fig. S7). Losses unique to the *Trypanosoma/Perkinsela* cluster are common (95 KEGG IDs fall into this category), as well as losses unique to photosynthetic euglenids (65 KEGG IDs; Additional file 8: Fig. S7). These results suggest that the clustering pattern recovered by UMAP reflects widespread convergent reduction in metabolic repertoires across several euglenozoan lineages.

Reduction of metabolic complexity in free-living organisms is not unprecedented, especially in multicellular ones with complex feeding behavior [61], but also in unicellular eukaryotes [62]. We speculate that certain eukaryotic lineages are predisposed to losses decreasing their metabolic capabilities, similarly to kinetoplastids and alveolates (exemplified by ciliates). These losses, in turn, might make the representatives of these lineages prone to switching to parasitic/symbiotic lifestyles. Below we describe the main evolutionary changes in euglenozoan metabolism. Of note, we intentionally refrain from discussing the subcellular localization of most of the enzymes mentioned in the following sections since we find such predictions unreliable without proteomic or other kinds of experimental evidence.

### Amino acid metabolism

**Fig. 3:**
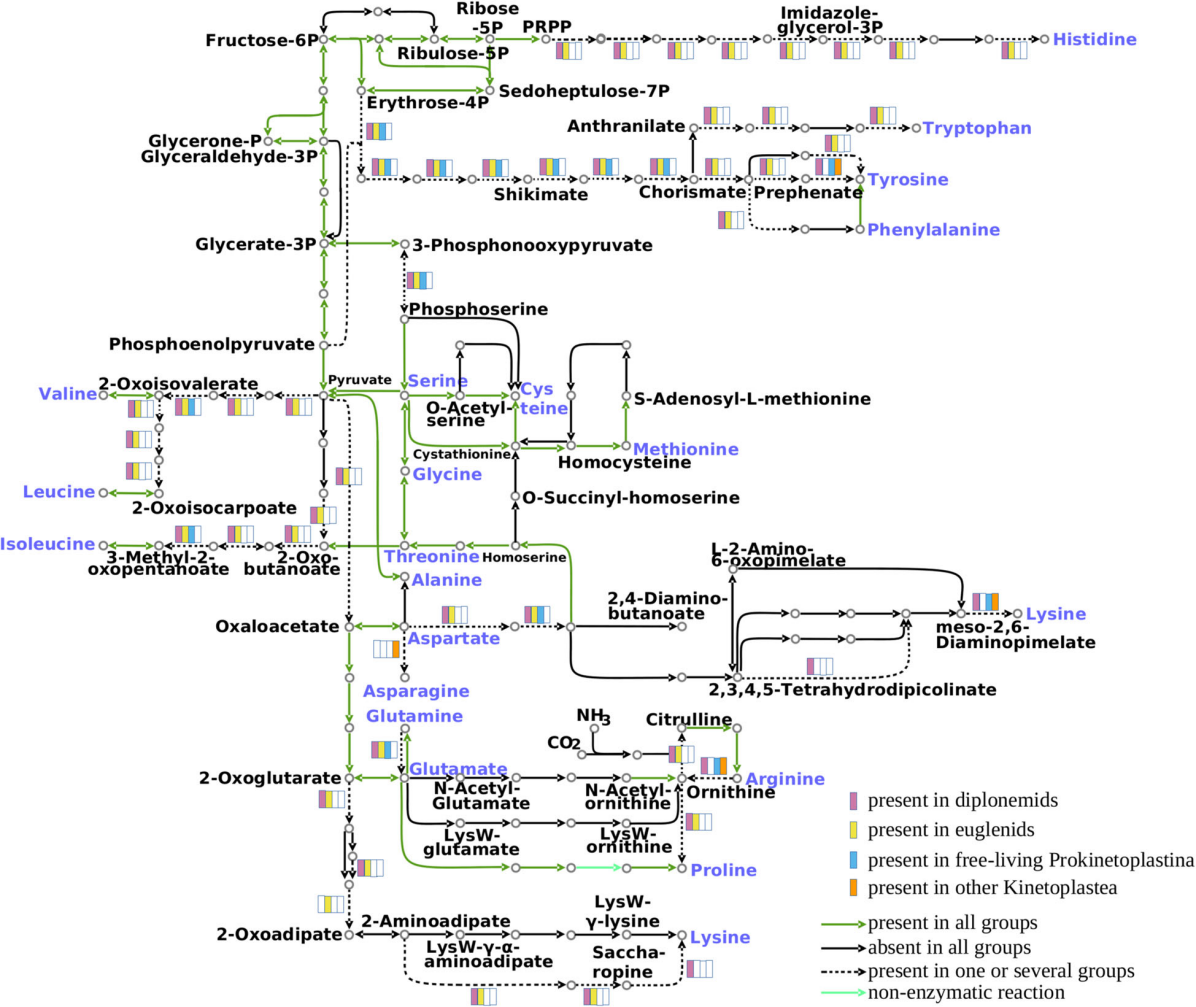
A map of amino acid metabolism in diplonemids, euglenids, and kinetoplastids. The 20 standard amino acids are shown in blue. The presence of enzymes is marked with purple, yellow, blue, and orange colors for diplonemids, euglenids, free-living prokinetoplastids, and other kinetoplastids, respectively. In the case that an enzyme is present in all three groups, the corresponding arrows are filled with green; in the case of its absence in all lineages, arrows are black; the presence in certain lineages only is marked with dashed arrows. The light green arrow represents a non-enzymatic reaction. A protein is considered as present in a group if it is identified in at least two species; for free-living prokinetoplastids, a presence of a gene is inferred if it is found in at least one species

Metabolically versatile diplonemids and euglenids are able to synthesize all 20 amino acids, like prokaryotes, plants, and some algae [61, 63], although a few biosynthetic enzymes could not be identified in euglenozoan transcriptomes. The reduction of amino acid biosynthetic capabilities is very often observed in heterotrophs preying on other organisms [61]. This, however, does not appear to be the case for diplonemids, which retain a full spectrum of genes encoding amino acid biosynthesis proteins. The possibility of obtaining amino acids from both biosynthetic pathways and food sources might be an important factor for the ecological success of diplonemids. Kinetoplastids appear to have undergone multiple losses of genes encoding enzymes of amino acid biosynthesis. In agreement with previous studies, all kinetoplastids appear to be auxotrophic for histidine, lysine, isoleucine, leucine, valine, phenylalanine, tryptophan, and tyrosine (Fig. 3; Additional file 9: Table S3) [59, 64]. Among kinetoplastids analyzed herein, only *L. pyrrhocoris* is capable of arginine biosynthesis from citrulline. The pathways for the biosynthesis of aromatic amino acids and histidine were lost from kinetoplastids. However, free-living prokinetoplastids, similarly to diplonemids and euglenids, still possess all the genes encoding proteins of the shikimate pathway, leading to the production of chorismate, a precursor of the aromatic amino acids, folate, and ubiquinone. As for the branched chain amino acid synthesis, only the enzyme catalyzing the last step of the pathway (branched chain amino acid aminotransferase) is encoded in kinetoplastid genomes (Additional file 9: Table S3). Its presence can be explained by the role it plays as the first enzyme of the branched chain amino acid catabolism, converting them to the corresponding ketocarboxylic acids [59]. In addition, prokinetoplastid PhF-6 carries a gene encoding dihydroxy-acid dehydratase, catalyzing the penultimate step of valine and isoleucine biosynthesis, which also participates in pantothenate biosynthesis.

The ability to synthesize glutamate from 2-oxoglutarate and glutamine was lost after neobodonids split, along with the ability to convert glutamate to 4-aminobutanoate (the first step of the γ-aminobutirate shunt). S-Methyl-5-thioribose kinase, an enzyme of the methionine recycling pathway, is present in diplonemids and some kinetoplastids (*P. confusum* and Leishmaniinae), but not in *B. saltans* and euglenids (Additional file 9: Table S4). However, the presence of methylthioadenosine phosphorylase may compensate for this loss. This enzyme is present in most euglenozoans except for *Perkinsela* and *B. saltans*, suggesting that these two species are not capable of recycling methionine.

Several proteins involved in amino acid metabolism in Euglenozoa are of interest from an evolutionary perspective. For example, euglenozoans appear to possess at least two different histidinol-phosphate phosphatases (HPPs), one (identified in *E. gracilis*) belonging to the family of inositol monophosphatase-like (IMP) proteins and the other (from prokinetoplastid PhF-6) to the polymerase and histidinol-phosphate phosphatase family. Euglenozoa are characterized by the presence of a penta-functional AROM protein, previously found only in prokaryotes, fungi, apicomplexa, ciliates, and oomycetes [65–67]. For a more detailed discussion on these proteins and additional information regarding particular amino acid biosynthetic enzymes, the reader is referred to Additional file 10 [68–93].

Euglenozoans utilize a number of amino acids as an energy source (Additional file 9: Table S5). The enzymes for the conversion of alanine, aspartate, asparagine, threonine, glutamate, histidine, branched chain amino acids (valine, leucine, and isoleucine), and proline to tricarboxylic acid (TCA) cycle intermediates or their precursors are present; thus, these amino acids can be readily used for energy metabolism and gluconeogenesis. Diplonemids can additionally generate energy from glutamine via a glutaminase. Threonine is not used as an energy source by Leishmaniinae or euglenids. The other euglenozoans oxidize threonine via the l-2-amino-3-oxobutanoate pathway, where threonine dehydrogenase acts in concert with 2-amino-3-ketobutyrate coenzyme A ligase in the formation of a TCA cycle intermediate, acetyl-CoA [94]. Histidine can be metabolized to glutamate only by *R. humris*, *S. specki*, free-living prokinetoplastids, *B. saltans*, *P. confusum*, and *T. grayi* (Additional file 9: Table S5).

### Nucleotide metabolism

The loss of the purine biosynthetic pathways was known for *B. saltans* and trypanosomatids and can now be extended to other kinetoplastids (Additional file 9: Table S6). Only one enzyme of the pathway, adenylosuccinate lyase, is encoded in their genomes/transcriptomes. Its presence in kinetoplastids is not surprising since it also functions in purine salvage as a part of the purine-nucleotide cycle (Additional file 9: Table S6). Diplonemids and phototrophic euglenids possess all the enzymes necessary for inosine monophosphate biosynthesis from phosphoribosyl diphosphate and glutamine, while the free-living bacteriovores such as bodonids and the parasitic trypanosomatids depend on their prey or their host for the provision of purines. In addition to the loss of the purine biosynthetic pathway, trypanosomatids lost xanthine oxidase and, thus, are unable to produce uric acid, a potent antioxidant, similarly to euglenids and *Perkinsela* sp.

In Euglenozoa, a number of enzymes play an important role in the salvage and interconversion of purine bases and their nucleotides (Additional file 9: Table S6). Previously, it was noticed that in trypanosomatids a few of these salvage enzymes are located within glycosomes [95]. A search for peroxisome-targeting sequences in bodonids, prokinetoplastids, and diplonemids revealed that numerous enzymes are also likely associated with glycosomes (Additional file 9: Table S6).

The biosynthesis of pyrimidines starts with uridine 5-monophosphate (UMP), which is synthesized by the subsequent action of six enzymes [96]. In Amoebozoa and Metazoa, the first three reactions are catalyzed by a trifunctional CAD protein [97, 98]. In fungi, a similar protein exists but lacks the dihydroorotase function, while in bacteria, archaea, and other eukaryotes, the first three reactions of the pathway are carried out by three separate proteins [96]. In trypanosomatids, these are also catalyzed by three independent enzymes, which in some species form multiprotein complexes [96, 99].

All Euglenozoa, except for *Perkinsela*, are capable of de novo pyrimidine biosynthesis (Additional file 9: Table S7). The first three enzymes of the pathway, carbamoyl-phosphate synthase, aspartate carbamoyltransferase, and dihydroorotase, were identified in almost all species and appear to be encoded by separate genes in all organisms analyzed, although it is unclear whether these proteins form a functional complex, similarly to the situation observed in *Trypanosoma cruzi* [96]. Trypanosomatids are known to possess a cytosolic, fumarate-dependent dihydroorotate dehydrogenase (DHODH), similar to yeast, while most other organisms carry a mitochondrial, ubiquinone-dependent enzyme [100, 101]. Our analyses demonstrate (although with a weak support) that in bodonids and trypanosomatids the gene for DHODH is of bacterial origin and encodes a soluble rather than a mitochondrial enzyme (Additional file 11: Fig. S8). Bacterial origin of the kinetoplastid DHODH was previously demonstrated by Annoura et al. [79] and is additionally supported by the fact that the trypanosomal enzyme can be inhibited by known inhibitors of bacterial DHODHs [80]. Most diplonemids and euglenids encode a mitochondrial ubiquinone-dependent enzyme, with the exception of *Hemistasia* and *Eutreptiella*, which have genes for both isofunctional enzymes. The origin of genes encoding fumarate-dependent DHODH in these organisms is likely different from that in kinetoplastids, as suggested by clustering of the respective sequences within a different clade of eukaryotic proteins. The acquisition of a soluble fumarate-dependent DHODH at the expense of the original mitochondrial isofunctional enzyme allows trypanosomatids and yeast to completely suppress the activity of their mitochondrial respiratory chain, without interference with their pyrimidine biosynthetic capacities [102]. These organisms are able to switch from oxidative phosphorylation to long-term glucose fermentation and under such conditions have a tendency to lose their mitochondrial DNA, forming dyskinetoplastic mutants [103–105]. It is currently unknown whether the other euglenozoans with a soluble DHODH share this capacity.

In trypanosomatids, the last step of the UMP biosynthesis is catalyzed by a glycosomal bifunctional enzyme comprised of orotate phosphoribosyltransferase (OPRT) and orotidine 5′-monophosphate decarboxylase (OMPDC) fused in the reverse order (pyrF/E, or OPRT/OMPDC) as compared to its metazoan counterpart called uridine-monophosphate synthase (UMPS; pyrE/F, or OMPDC/OPRT) [99]. Euglenozoa show a complex pattern of UMP synthesis, since we have identified separate transcripts encoding *pyrF* and *pyrE* genes, as well as their fusions (Additional file 9: Table S7). Although full genome sequences and a detailed phylogenetic analysis are necessary in order to infer the evolutionary history of these genes, it is likely that the common ancestor of euglenozoans possessed two separate genes (as in free-living prokinetoplastids). They possibly underwent duplications and subsequent fusions in either direct (*pyrE/F*; *E. gracilis* and *E. gymnastica*) or reverse order (*pyrF/E*; trypanosomatids, bodonids, and *H. phaeocysticola*), with the subsequent loss of one or both original genes. Finally, in trypanosomatids and bodonids, the bifunctional protein is targeted to glycosomes (Additional file 9: Table S7).

Important enzymes of the pyrimidine metabolism were lost in kinetoplastids. For example, trypanosomatids and *Perkinsela* lost enzymes of reductive uracil degradation and thymine degradation (Additional file 12: Fig. S12A). DCMP deaminase, an important contributor to the dTTP biosynthesis through deamination of dCMP to dUMP in many eukaryotes, was apparently lost after the Prokinetoplastina split point.

### Fatty acid biosynthesis

Trypanosomatids rely on a set of integral membrane elongases for fatty acid (FA) biosynthesis, using butyryl-CoA as a primer [106, 107]. Animals and fungi use these proteins to extend saturated and unsaturated FAs, while employing a multidomain fatty acid synthase I for bulk FA biosynthesis [108–110]. Trypanosomatids lack FAS I homologue but share with opisthokonts the presence of a mitochondrial FAS II system [107, 111]. *E. gracilis* is known to possess FAS I system in the cytosol and FAS II in the mitochondria and chloroplasts [112–114]. We have identified putative elongases in all organisms except *Perkinsela* sp. (Additional file 9: Table S8). Preliminary phylogenetic analysis suggests that diplonemids, euglenids, and bodonids possess homologues of trypanosomatid polyunsaturated FA (PUFA) elongases (TbELO4, LmE1, and E2 in Additional file 9: Table S8) [106, 115]. The remaining euglenozoan proteins show phylogenetic affiliation to conventional elongases from several reference species (see the “Materials and methods” section). Although full-length FAS I proteins are absent from the transcriptome assemblies, the identification of FAS I candidates in several diplonemids and euglenids suggests that they use a conventional FAS I in contrast to obligatory parasitic Trypanosomatidae. The situation with other kinetoplastids is less clear, since partial transcripts carrying more than three FAS I domains were identified only in *A. hoyamushi* and *T. borreli*. Mitochondrial FAS appears to be present in all studied euglenozoans (Additional file 9: Table S8).

### Cofactors and vitamins

As previously reported [59], kinetoplastids are capable of riboflavin and flavin mononucleotide interconversions, similarly to diplonemids and euglenids. The latter two groups, as well as the free-living Prokinetoplastina spp., are able to synthesize riboflavin de novo, since all the required enzymes are present in their transcriptomes (Additional file 12: Fig. S12B). The only exception is a single protein, 5-amino-6-(5-phospho-D-ribitylamino) uracil phosphatase, catalyzing dephosphorylation of 5-amino-6-(5-phosphoribitylamino) uracil, which was not identified in euglenids.

Diplonemids, euglenids, and free-living prokinetoplastids can synthesize tetrahydrobiopterin (BH_4_) from GTP (Additional file 12: Fig. S12C). The first enzyme of the pathway, GTP cyclohydrolase I, catalyzing the conversion of GTP to dihydroneopterin triphosphate, is present in the transcriptome and the draft genome of *T. borreli* [47]. In addition, diplonemids, euglenids, and free-living prokinetoplastids appear to be capable of folate biosynthesis from chorismate and GTP, while other kinetoplastids only perform folate-pool interconversions (Additional file 12: Fig. S12C) [116]. Diplonemids and euglenids can synthesize thiamine (Additional file 12: Fig. S12D) and convert pyridoxin (vitamin B_6_) to pyridoxal phosphate, similarly to bodonids and free-living Prokinetoplastina spp. Diplonemids, euglenids, and PhF-6 can synthesize pantoic acid from pyruvate.

### Digestion of bacterial cell walls

The gene for a bacterial-type N-acetylmuramate 6-phosphate etherase [117] involved in the delactoylation of a cell wall murein constituent N-acetylmureate 6-phosphate, which is required for the degradation of murein, has been identified in a number of free-living protists, such as *N. gruberi*, but was not detected in trypanosomatids. However, it was recently found in *B. saltans* [59] and here in *N. designis*, free-living prokinetoplastids, and *H. phaeocysticola* (Additional file 9: Table S9). The d-lactate released by the action of etherase mentioned above and d-alanine liberated by peptidases from the cell walls of prey bacteria are further metabolized by d-lactate dehydrogenase (LDH) and alanine racemase, as the respective genes were identified in the three major euglenozoan lineages. Our phylogenetic analysis (Additional file 11: Fig. S9), although with weak support due to high sequence divergence, is in agreement with the results of Nývltová et al. [118] suggesting multiple LGT events between eukaryotes and prokaryotes in the evolutionary history of genes encoding LDH.

### NADPH-dependent thiol-redox systems

Most organisms rely on glutathione and thioredoxin NADPH-dependent disulfide reductase systems for oxidative stress protection, cell signaling, DNA replication, metal homeostasis maintenance, detoxification of xenobiotics, and other purposes [119–121]. These thiol-redox systems have largely overlapping functions, and therefore, loss of one or even both of them is not unprecedented [120, 122]. Alternative antioxidant systems are also described and can be exemplified by the ones based on mycothiol or bacilithiol in various bacteria, phytochelatins in plants, and ovothiol in sea urchins [123–126]. Trypanosomatids are known to have developed a minimalistic thiol-redox system based solely on trypanothione [40, 127, 128]. The presence of trypanothione reductase, and thus, the ability to utilize trypanothione, was also demonstrated for *E. gracilis* and *B. saltans* [59, 129]. These observations and virtual lack of information on the distribution of trypanothione and other thiol-redox systems in Euglenozoa, except for trypanosomatids, eubodonids, and *E. gracilis*, prompted us to investigate this subject.

**Fig. 4:**
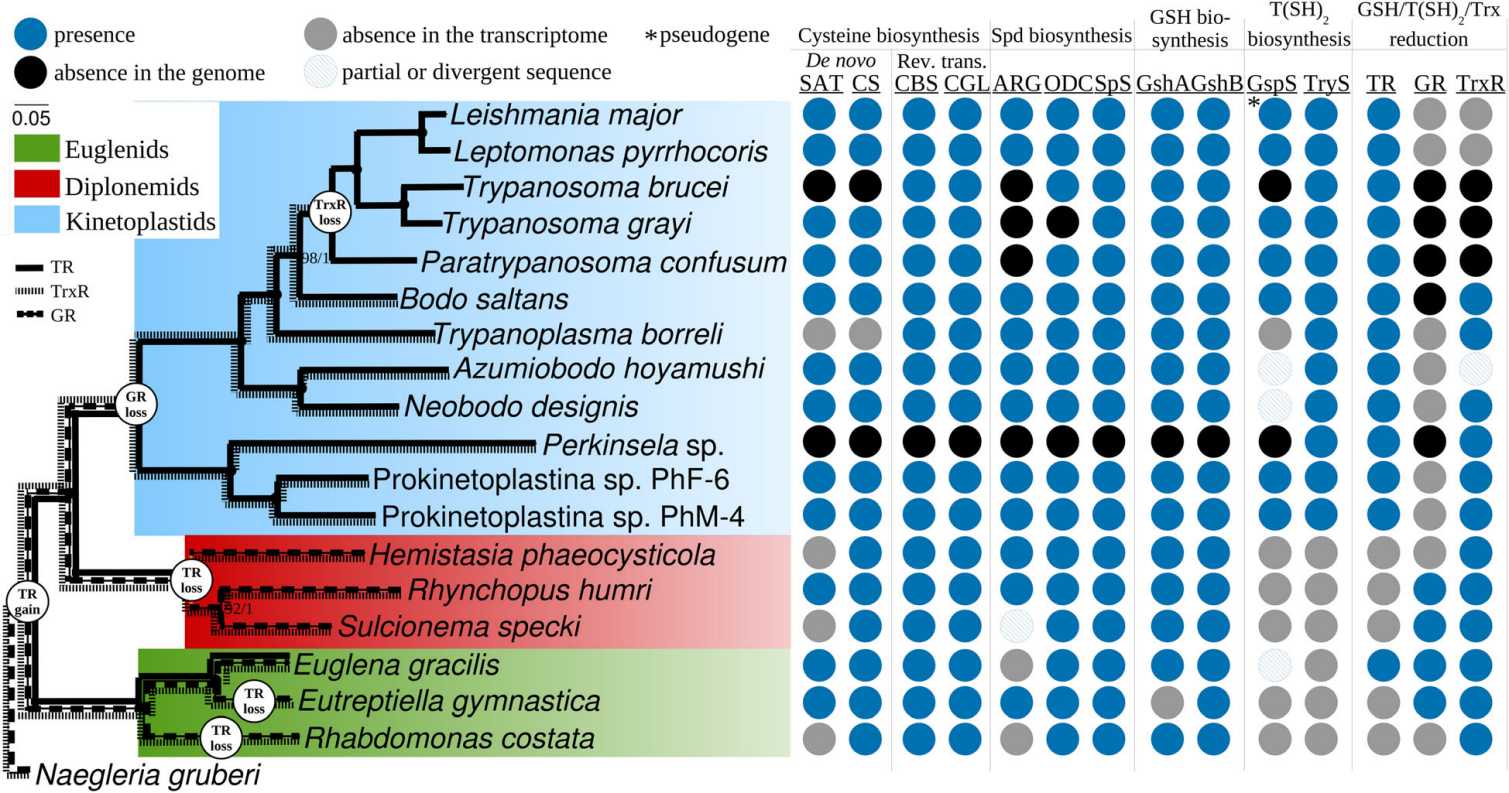
Presence/absence patterns of the proteins involved in the trypanothione biosynthesis and utilization in Euglenozoa. Gene presence, absence from a genome, absence from a transcriptome, and presence of a partial/divergent sequence are indicated with blue, black, gray, and blue hatched circles, respectively. Putative evolutionary gain/loss events of three thiol-redox systems in Euglenozoa are shown with white circles. Enzymes’ abbreviations are as follows: ARG, arginase; CBS, cystathionine β-synthetase; CGL, cystathionine γ-lyase; CS, cysteine synthetase; GR, glutathione reductase; GshA, γ-glutamyl-cysteine synthetase; GshB, glutathione synthetase; GspS, glutathionylspermidine synthetase; ODC, ornithine decarboxylase; SAT, serine acetyltransferase; SpS, spermidine synthetase; TR, trypanothione reductase; TrxR, thioredoxin reductase; TryS, trypanothione synthetase. Rev. trans., reverse trans-sulfuration pathway

Trypanothione is a low molecular weight thiol composed of two glutathione molecules joined by a spermidine linker [40]. In turn, glutathione is a tripeptide consisting of cysteine, glutamate, and glycine [130]. Glutamate is readily available for trypanosomatids in the insect midgut, while glycine can be synthesized de novo from serine [59]. There are two main pathways leading to the formation of cysteine: de novo biosynthesis from serine and a reverse *trans*-sulfuration pathway (RTS). All euglenozoans in our dataset, except for *T. brucei*, *T. borreli*, and *Perkinsela*, are capable of de novo cysteine biosynthesis (Fig. 4; Additional file 9: Table S10). The three latter organisms lack both enzymes of the pathway, serine acetyltransferase and cysteine synthetase. *T. brucei* possesses efficient cysteine transporters enabling acquisition of this amino acid from the vertebrate’s bloodstream [131]. *Perkinsela* uptakes glutathione and spermidine from the cytoplasm of its amoebozoan host, preserving only trypanothione synthetase (TryS) out of all enzymes involved in trypanothione biosynthesis [30]. *T. borreli* either can uptake cysteine from the fish blood or relies entirely on the RTS system, since both enzymes of the pathway, cystathionine β-synthetase and cystathionine γ-lyase, are present in its transcriptome, similarly to the situation observed in other species in the dataset. Both enzymes of the glutathione biosynthesis, γ-glutamyl-cysteine synthetase and glutathione synthetase, were readily identified in all analyzed genomes and transcriptomes, except for *Perkinsela* (Fig. 4; Additional file 9: Table S10).

Besides glutathione, the polyamine spermidine represents another molecule necessary for trypanothione biosynthesis. All three enzymes necessary for the spermidine biosynthesis from arginine were identified in diplonemids, a euglenid *E. gymnastica*, in Prokinetoplastina, neobodonids, *T. borreli*, and *B. saltans*. Consistent with previous studies, our results suggest that among trypanosomatids only representatives of the subfamily Leishmaniinae possess arginase, which metabolizes arginine into ornithine and urea at the first step of the trypanothione biosynthesis, while other species apparently uptake ornithine from the host [59]. *T. grayi*, in addition to arginase, also lacks ornithine decarboxylase and, similarly to *T. cruzi*, can potentially scavenge polyamines from the host [132, 133]. The transcriptome of *E. gracilis* does not encode arginase. Instead, this protist can produce ornithine via the arginine dihydrolase pathway [134, 135].

**Fig. 5:**
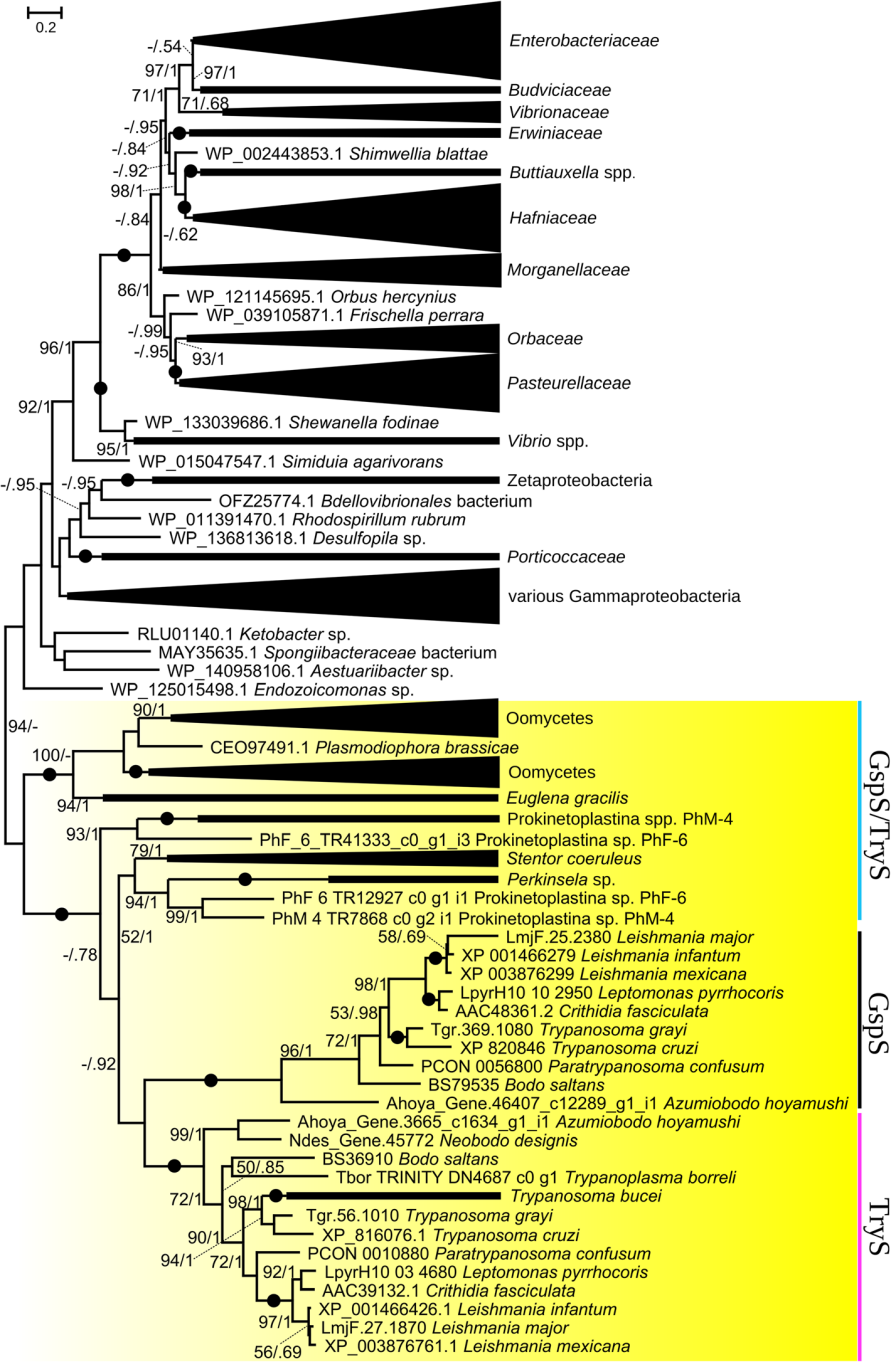
A phylogenetic tree of glutathionylspermidine (GspS) and trypanothione synthases (TryS) based on a trimmed alignment of 363 amino acids. Nodes exhibiting maximal bootstrap support and posterior probability (PP) are marked by black circles. Only bootstrap supports ≥ 50 and PP values ≥ 0.5 are shown. Eukaryotic sequences are shown on yellow background

In trypanosomatids, the two-step addition of glutathione to amino groups of spermidine yielding trypanothione is carried out either by TryS or requires a subsequent action of glutathionylspermidine synthetase (GspS) and TryS [127, 136]. All kinetoplastids within our dataset carry TryS, while GspS was not identified in *T. brucei*, *T. borreli*, and *Perkinsela*, and the respective gene is pseudogenized and carries two in-frame stop codons in the genome of *L. major* [137]. Trypanosomatid and bodonid GspS and TryS sequences form two distinct, well-supported clades on maximum-likelihood and Bayesian trees (Fig. 5). Interestingly, the homologous proteins in Prokinetoplastina and *E. gracilis* fall into different clades, which also contain a few sequences identified in the ciliate *Stentor coeruleus*, the rhizarian *Plasmodiophora brassicae*, and several oomycetes [138].

**Fig. 6:**
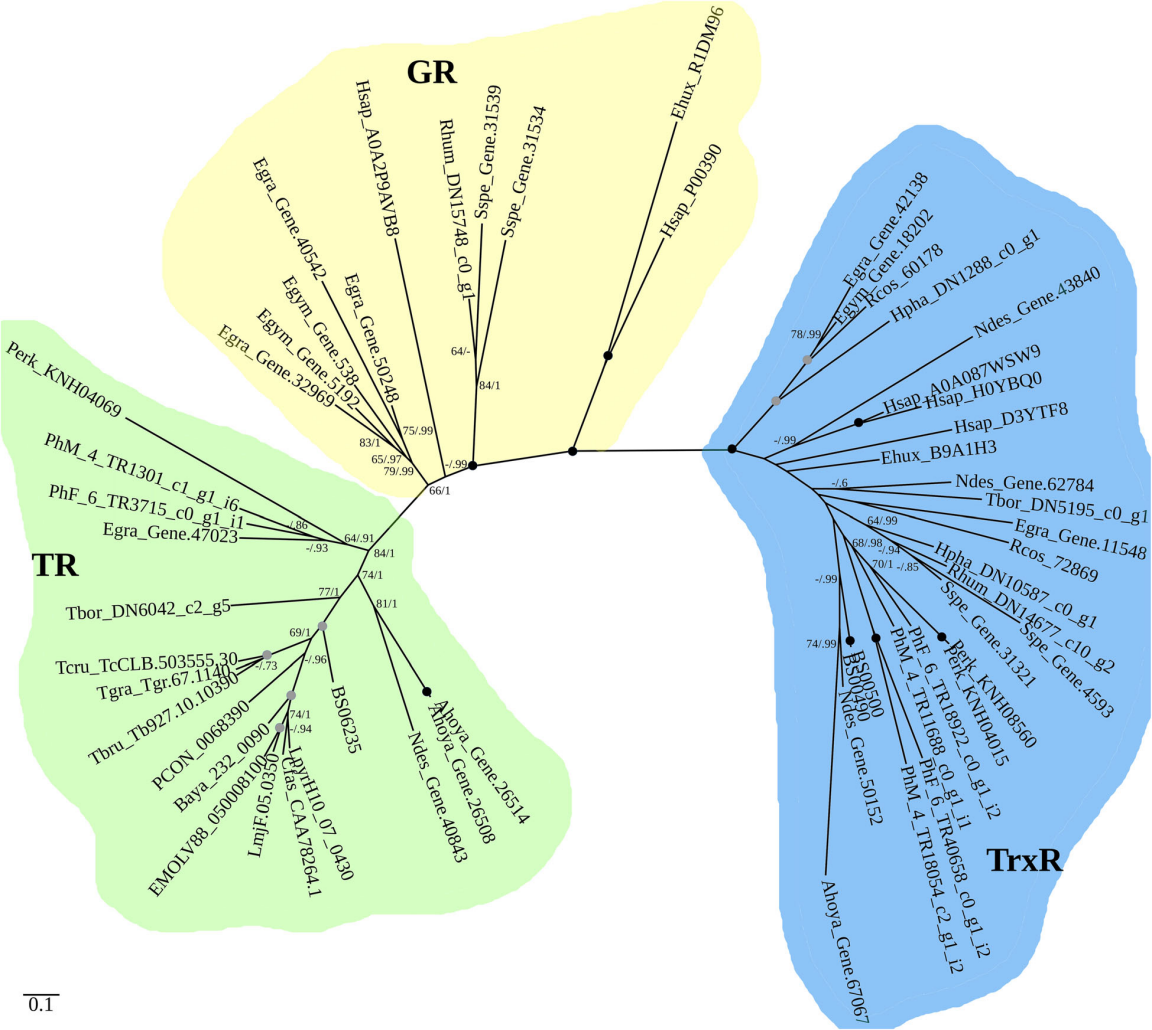
A phylogenetic tree of trypanothione (TR; green background), thioredoxin (TrxR; blue), and glutathione reductases (GR; yellow) based on a trimmed alignment of 352 amino acids. Nodes exhibiting maximal bootstrap support and posterior probability (PP) are marked by black circles; gray circles signify ≥ 90% bootstrap support and PP ≥ 0.9. Only bootstrap support and PP values ≥ 0.6 are shown. Species abbreviations in protein IDs are as follows: Ahoya, *Azumiobodo hoyamushi*; Baya, *Blechomonas ayalai*; BS, *Bodo saltans*; Cfas, *Crithidia fasciculata*; Ecoli, *Escherichia coli*; Egra, *Euglena gracilis*; Ehux, *Emiliania huxleyi*; EMOLV, *Endotrypanum monterogeii*; Egym, *Eutreptiella gymnastica*; Hpha, *Hemistasia phaeocysticola*; Hsap, *Homo sapiens*; Linf, *Leishmania infantum*; LmjF, *Leishmania major*; Lmex, *Leishmania mexicana*; Lpyr, *Leptomonas pyrrhocoris*; Ndes, *Neobodo designis*; PCON, *Paratrypanosoma confusum*; Perk, *Perkinsela* sp.; PhF_6, prokinetoplastid species PhF-6; PhM_4, prokinetoplastid species PhM-4; Rhum, *Rhynchopus humris*; Rcos, *Rhabdomonas costata*; Sspe, *Sulcionema specki*; Tbor, *Trypanoplasma borreli*; Tbru, *Trypanosoma brucei*; Tcru, *Trypanosoma cruzi*; Tgra, *Trypanosoma grayi*

BLAST and HMM-based searches for putative trypanothione reductases (TRs) resulted in the identification of related glutathione (GR) and thioredoxin (TrxR) reductases. Additional searches using these as queries (see the “Materials and methods” section) identified sequences subsequently used for building a phylogenetic tree (Fig. 6). Putative TR sequences were found in all studied kinetoplastids and *E. gracilis*. TR from *E. gracilis* clusters with the putative TRs of Prokinetoplastina. TrxRs were absent only from all trypanosomatids analyzed here (Figs. 4 and 6), while GRs appear to be restricted to diplonemids and euglenids.

Thus, the evolution of NADPH-dependent disulfide reductase systems may serve as an example of a gradual loss of metabolic capabilities in kinetoplastids. The most parsimonious evolutionary scenario implies that a common ancestor of euglenids, diplonemids, and kinetoplastids possessed glutathione, thioredoxin, and glutathionylspermidine/trypanothione-based NADPH-dependent disulfide reductase systems (Fig. 4). For unknown reasons, all three systems were retained only in the genus *Euglena* (including a non-photosynthetic *Euglena longa* with TR sequence 87% identical to that of *E. gracilis*), while other euglenids and diplonemids have lost the ability to synthesize trypanothione and the respective trypanothione utilization enzyme, TR. The loss of *GR* and *TrxR* occurred in the common ancestors of kinetoplastids and trypanosomatids, respectively, likely using glutathione mainly as a precursor for the trypanothione biosynthesis [128]. Manta et al. hypothesized that the euglenozoan ancestor might have gained *GspS* from a bacterium by LGT, which then gave rise to *TryS* by gene duplication and neofunctionalization [127]. Our findings suggest that either there were several events of LGT from bacteria to eukaryotes (at least in euglenozoans, oomycetes, ciliates, and rhizarians) or *GspS* was an ancestral gene present in the last eukaryotic common ancestor and was subsequently lost multiple times during the evolution of eukaryotes (Fig. 5).

Trypanothione is characterized by higher interaction with macrophage-derived reactive nitrogen species than glutathione, and the dithiolic nature and physiochemical properties of the former make it more efficient and flexible in the reduction of dehydroascorbate and ribonucleotide reductase [127, 128, 139]. The advantages of the trypanothione system over glutathione and thioredoxin, combined with the trend for general gene loss observed in kinetoplastids, made two latter antioxidant defense systems dispensable and facilitated a gradual loss of *GR* and *TrxR*. The same trend is observed with the loss of *GspS* and its pseudogenization in some trypanosomatids, where its role remains uncertain since the trypanothione biosynthesis is carried out by TryS [140].

### Composition of the DNA pre-replication complex

In eukaryotes, DNA replication is invariably initiated by assembly on replication origins of the pre-replication complex (pre-RC) typically consisting of four main components: the origin recognition (ORC), minichromosome maintenance complexes (MCM), and CDC6 and Cdt1 proteins [141]. The activity of the pre-RC is initiated by the CDC6-modulated activity of the ORC, which upon binding to the replication origin engages MCM, with the help of Cdt1 [142, 143].

In Trypanosomatidae, the composition of the heterohexameric MCM complex resembles that of other eukaryotes [37], while the situation with other components of pre-RC is totally different. For a long time, only one putative ORC subunit (ORC1/CDC6) has been known, which led to a false conclusion that trypanosomatids possess an archaeal-like single-protein or homomeric pre-RC [144]. However, a few more proteins possibly acting during the initiation of replication were subsequently identified, although only two of them, which are remote homologues of the eukaryotic ORC1 and ORC4, were demonstrated to interact with the components of pre-RC in *T. brucei* [37]. The composition of pre-RC in Euglenozoa beyond Trypanosomatidae remained unstudied.

**Fig. 7:**
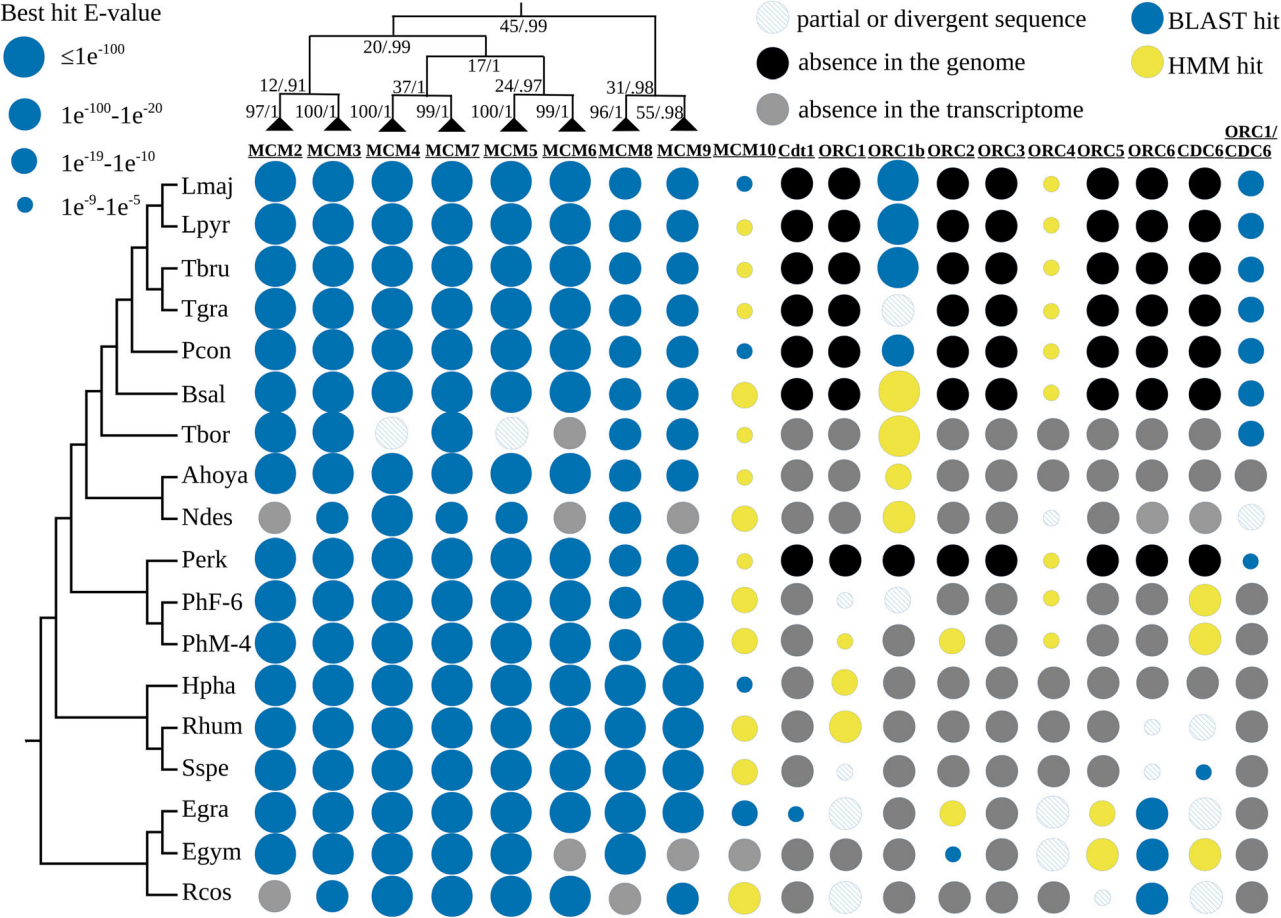
Presence/absence patterns of the components of the pre-replication complex in Euglenozoa. The presence of proteins identifiable by BLAST or by HMM-based searches only is shown in blue and yellow, respectively. The presence of a partial or divergent sequence is marked with hatched circles. Circle sizes are proportional to BLAST or HMM E values for corresponding hits. Protein absence in a genome or transcriptome is indicated with black- and gray-filled circles, respectively. A cladogram for the MCM complex subunits is based on maximum-likelihood (LG+F+I+G4, 1000 bootstrap replicates) and Bayesian (WAG+I+G4, one million generations) phylogenetic trees. Numbers at the nodes represent bootstrap supports and posterior probabilities. The species cladogram is based on Fig. 1. Abbreviations: MCM, minichromosome maintenance complex; ORC, origin recognition complex; CDC6, cell division cycle 6; Cdt1, CDC10-dependent transcript 1.

In our study, most subunits of the replicative helicase (MCM2-7), belonging to the AAA+ protein family (ATPases associated with a variety of activities), appear to be the least divergent compared to the opisthokont homologues and were readily identified by BLAST in all euglenozoan genomes and transcriptomes analyzed, with few exceptions (Fig. 7; Additional file 9: Table S11; Additional file 10). We assume that the patchy distribution of some MCM subunits in our dataset shall be attributed to low levels or lack of expression, rather than to a genuine absence of the corresponding genes.

The identification of the remaining pre-RC components (six ORC subunits, CDC6 and Cdt1) was much more challenging (Fig. 7; Additional file 9: Table S11; Additional file 10). Although identified in all three strains of *E. gracilis* analyzed, Cdt1 appears to be absent, not expressed, or highly divergent in all other euglenozoans. A search for the winged-helix initiator protein, representing a functional analogue of the eukaryotic Cdt1 in Archaea [142], also did not yield any euglenozoan hits. The fact that the homologues of Cdt1 were not identified in any euglenozoan except for *E. gracilis* is not surprising given its poor conservation even in eukaryotes well-studied in this respect, its putative absence in several other protist groups [145], and Cdt1’s generally highly variable length [146] and lack of well-defined domains [147].

The search for the ORC subunits and CDC6 protein led us to a conclusion that the pre-RC of euglenids resembles the classical eukaryotic structure the most, as only one protein out of six ORC components (ORC3) was not identified in their transcriptomes. In contrast, diplonemids carry the most divergent machinery with only ORC1 and CDC6 confidently identified, in addition to the MCM sequences mentioned above. While all kinetoplastids possess putative homologues of ORC1/CDC6, ORC4, and kinetoplastid-specific ORC1b, two variants of ORC1 and CDC6 were found in Prokinetoplastina. The identification of weak hits to ORC1, ORC2, and CDC6 in the early branching kinetoplastids leads us to conclude that these proteins are rather highly divergent than lost altogether. Still, a loss or a non-orthologous displacement of some elements of the replication machinery, as observed in Archaea [75], cannot be ruled out at this point.

While the reasons for such a divergence of the pre-RC in Euglenozoa remain unclear, we speculate that it is related to their omnipresent polycistronic transcription [148, 149]. In the apparent absence of transcriptional regulation in these flagellates, additional requirements are likely imposed to avoid clashes between replication and transcription. Moreover, some trypanosomatids are characterized by mosaic aneuploidy [150], which complicates matters even further. The peculiarities of replication and transcription might have accelerated evolution of the pre-RC elements, as well as the means by which the activity of Cdt1 is regulated. Interestingly, this protein is required for both DNA replication and chromosome segregation in humans, where it stabilizes kinetochore–microtubule attachments via interactions with the NDC80 kinetochore complex [151]. Since the latter also appears to be highly divergent in diplonemids and kinetoplastids, we speculate that the enhanced rates of evolution of their kinetochore machinery might have influenced the degree of conservation of the pre-RC elements.

### Kinetochore elements

The kinetochore is a modular multiprotein assemblage directing chromosome segregation during mitosis and meiosis [152, 153]. Kinetochores mediate interaction between spindle microtubules and centromeric DNA and are comprised of ~ 80 proteins in opisthokonts [92, 154]. The outer kinetochore, which is the most conserved part of the machinery that directly binds microtubules, is usually composed of complexes Ndc80, Mis12, and Knl1, forming a so-called KMN network [92]. The inner kinetochore is assembled on centromeres, which are in most organisms defined epigenetically via deposition of a centromere-specific histone H3 (cenH3) and peculiar histone modifications [155].

In all eukaryotes studied in this respect, proteins of the outer kinetochore directly interact with microtubules built from highly conserved α- and β-tubulins [156, 157]. However, while losses of certain components are not uncommon [158], the situation with the inner kinetochore is quite different. A set of over a dozen proteins interacting with the centromeric nucleosomes in vertebrates and yeast is referred to as the constitutive centromere-associated network (CCAN) [92]. The majority of CCAN proteins, except for cenH3-binding CenpC, is extremely divergent or even absent in numerous eukaryotic lineages [158, 159]. High divergence rates of centromeric DNA sequences and interacting inner kinetochore components can possibly be explained by the meiotic drive hypothesis in the case of organisms with asymmetric meiosis, where certain centromeric satellites can be advantageous for chromosome’s inclusion into an oocyte during female meiosis [160].

While most organisms retain at least some of the core kinetochore components described above, none of them could be identified in trypanosomatids, which instead harbor 20 unconventional kinetoplastid kinetochore proteins (KKTs) [38, 161]. These proteins are thought to form functional complexes, similarly to the situation observed in conventional kinetochores [162]. Additional sensitive HMM-based searches led to the identification of a remote Ndc80/Nuf2 homologue, suggesting that KKTs might not be as unconventional chromosome segregation machinery as originally thought. The present scenario postulates that these kinetoplastid-specific elements of the inner kinetochore are analogous to CCAN of vertebrates and fungi, while the elements of the outer kinetochore are widely distributed across eukaryotes, at the same time exhibiting variable levels of sequence divergence [163]. Moreover, the microtubule-binding activity of the BRCT-domain-containing KKT4 and its localization to the inner kinetochore suggests that kinetoplastids might possess a uniquely structured molecular machinery for chromosome segregation, while the exact function and localization of the distant Ndc80/Nuf2 homologue in these species remains to be elucidated [164].

Nearly nothing is known about the kinetochore composition in Euglenozoa except for trypanosomatids, *B. saltans* and *Perkinsela* [38, 162]. There are contradictory reports regarding the presence of the KKT proteins in *E. gracilis*. One group claimed to identify putative homologues of KKT10 and KKT19 in its genome [15], while other could not find such homologues in its transcriptome [162].

**Fig. 8:**
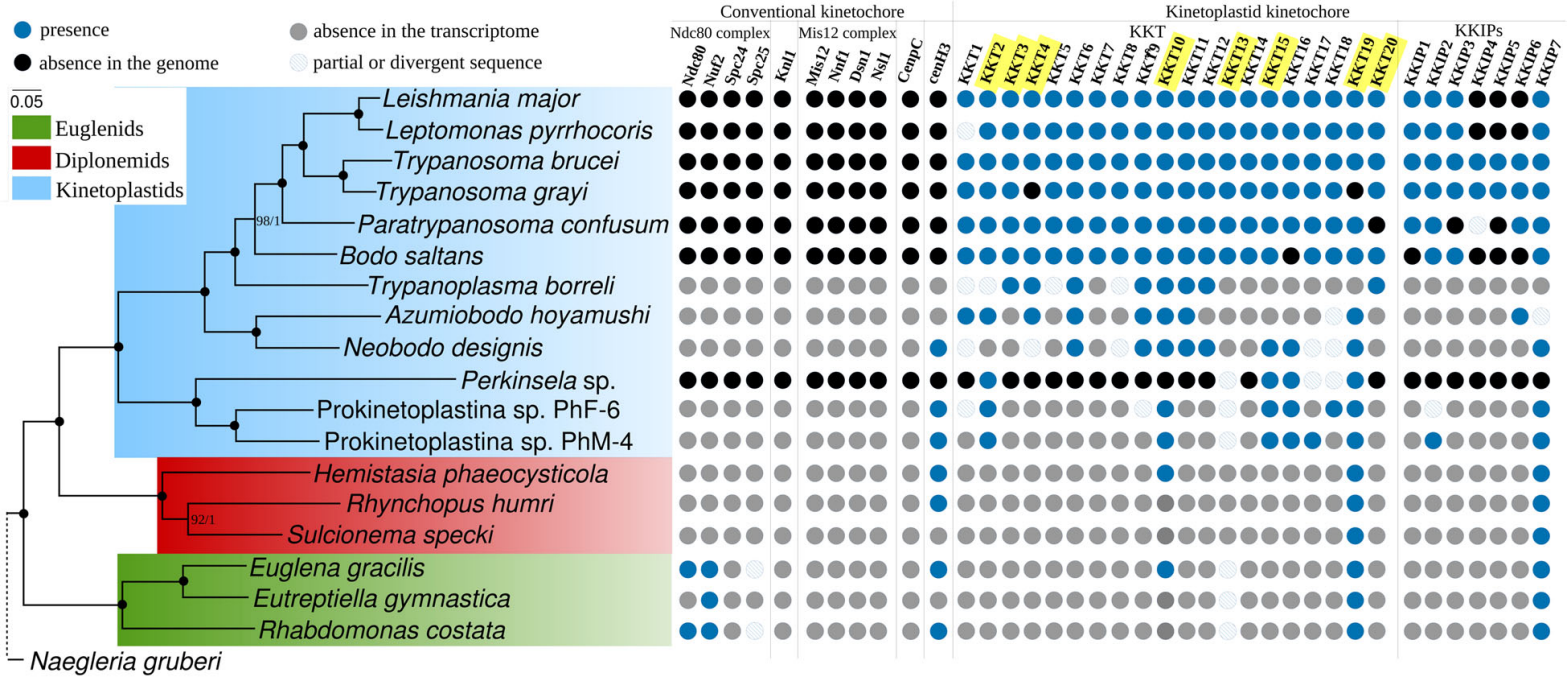
Presence/absence patterns of the components of the kinetochore machinery in Euglenozoa. Proteins’ presence, absence from a genome, absence from a transcriptome, and presence of a partial/divergent sequence are indicated with blue, black, gray, and blue hatched circles, respectively. KKT proteins possessing recognizable domains are marked in yellow. Abbreviations are as follows: cenH3, the centromeric variant of histone H3; CenpC, centromere-associated protein C; Dsn1, dosage suppressor of NNF1; KKIPs, kinetoplastid kinetochore-interacting proteins; KKTs, kinetoplastid kinetochore proteins; Knl1, kinetochore scaffold 1; Ndc80, nuclear division cycle 80; Nnf1, necessary for nuclear function 1; Nsl1, Nnf1 synthetic lethal; Nuf2, nuclear filament-containing protein 2; Spc24, spindle pole body component 24 homologue; Spc25, spindle pole body component 25 homologue

A search for a set of 20 KKTs (see the “Materials and methods” section and Additional file 10 for details) led to the identification of all respective homologues in trypanosomatids, with only a few exceptions (Fig. 8; Additional file 9: Table S12). A majority of KKTs was also detected in at least two bodonid genomes/transcriptomes, and only half of them were found in prokinetoplastids. In addition to Kinetoplastea, easily identifiable KKT10 and 19 homologues were found in both diplonemids and euglenids, which otherwise seem to lack KKTs (Fig. 8). Highly divergent sequences of the forkhead-associated (FHA) domain-containing KKT13 protein were identified in euglenids (Fig. 8; Additional file 9: Table S12; Additional file 10).

Our analyses suggest that other components of unconventional kinetochore, kinetoplastid kinetochore-interacting proteins (KKIPs), are restricted to kinetoplastids, apart from the phosphatase domain-containing KKIP7 readily identifiable in euglenids and diplonemids (Fig. 8). Some of the KKIP proteins are genus-specific within trypanosomatids (Additional file 10). Overall, KKT and KKIP sequences appear to be fast-evolving, since even the best-scoring hits are characterized by high p-distances to the reference trypanosomatid proteins (Additional file 13: Tables S13-S38).

Thus, out of 20 KKTs and 7 KKIPs, only putative homologues of KKT10, 13, 19, and KKIP7 could be identified outside of kinetoplastids. Importantly, euglenozoan hits to the trypanosomatid KKT sequences extend beyond the predicted domain borders, with most candidates yielding best reciprocal hits to the corresponding trypanosomatid sequences. However, the functions of these proteins remain to be elucidated experimentally, since we cannot exclude the possibility that they play a different role in Euglenozoa and were recruited to the kinetoplastid kinetochore.

Since our search for unconventional kinetochore proteins yielded only a few hits with non-specific domains outside kinetoplastids, the next step was to check for the presence of the most conserved elements of the conventional machinery. The presence of cenH3 is a defining trait for functional centromeres in all organisms studied in this respect, apart from trypanosomatids, holocentric insects, and certain stages during the development of *Caenorhabditis elegans* [165]. Following homology searches, we have delineated putative cenH3 sequences based on the presence of insertion in the loop 1 of the histone fold domain, divergent N-terminal tail, the absence of a conserved glutamine in the α1 helix of the histone fold domain, and a high level of divergence in the histone fold domain (Additional file 13: Table S39) [160]. In agreement with previous studies, no candidate cenH3 sequences were identified in trypanosomatids [166], while the transcriptomes of diplonemids (*H. phaeocysticola* and *R. humris*), euglenids (*E. gracilis* and *R. costata*), a neobodonid *N. designis*, and two prokinetoplastids PhF-6 and PhM-4 encode putative cenH3 sequences (Fig. 8; Additional file 9: Table S40). CenpC represents a direct interaction partner of cenH3 in opisthokonts, and thus, its presence/absence might serve as an indirect evidence for the presence/absence of cenH3 [167]. However, we could not identify this protein in any euglenozoans within our dataset (Fig. 8). Our search for the most conserved elements of the outer kinetochore resulted in the identification of Ndc80 components, including putative homologues of Nuf2 in all euglenids, Ndc80 and Spc25 in *E. gracilis* and *R. costata*, with Spc24 being absent from the whole dataset along with Mis12 complex and Knl1 protein (Fig. 8).

Overall, our results suggest that euglenids possess the most conserved outer kinetochore machinery among euglenozoans, which is assembled on centromeric chromatin via interactions with cenH3. The outer kinetochore either directly interacts with the centromere or the interaction is mediated by highly divergent or yet undescribed proteins. It is currently unknown whether the putative orthologues of KKTs and KKIPs identified in diplonemids and euglenids represent genuine kinetochore elements. In any case, diplonemids appear to possess a highly unusual kinetochore machinery yet to be described which, however, most likely requires cenH3 for its assembly. It suggests highly unusual chromosome segregation and/or cell division process in these marine protists.

### Analysis of the kinetochore and implications for the evolution of protein complexes in Euglenozoa

We speculate that the kinetochores in euglenozoans nicely illustrate a pattern observed also for other protein assemblages in this eukaryotic lineage. Only a few conventional protein complex subunits are retained, while others are either replaced by new elements with analogous and/or novel functions, or diverged beyond recognition. For example, trypanosomatid respiratory complexes possess a significant number of lineage-specific subunits along with at least several core elements shared with opisthokonts. The respiratory complexes of *T. brucei* incorporate from three (complex III) to over 30 (complex I) additional subunits, many of which were thought to be parasitism-related trypanosomatid-specific components, but later were identified in free-living *E. gracilis* and, thus, are now considered Euglenozoa-specific [168–170]. The respiratory complex I in *Diplonema papillatum* incorporates 15 diplonemid-specific subunits along with a set of universal and highly conserved eukaryotic components [171]. Information about the composition of other respiratory complexes in diplonemids is still fragmentary [169]. A homologue of basalin, which plays an important role in basal plate formation and which was thought to be restricted to *Trypanosoma* spp., yet was recently identified in *Leishmania* spp. using synteny analysis, serves as another example of rapid evolution [172]. In the mitochondrial protein import machinery of *T. brucei*, non-homologous proteins with analogous functions form complexes with only a few subunits shared with yeast [39]. The cristae-building MICOS complex may serve as yet another example of a protein machinery highly conserved across eukaryotes that within this protist lineage acquired subunits lacking homologues outside kinetoplastids [173]. Finally, approximately half of the mitochondrial ribosomal proteins in *T. brucei* are restricted to this and related species [174].

After the replacement of conventional protein complex subunits and/or recruitment of novel ones, the remaining core proteins co-evolve with these new interaction partners, becoming increasingly divergent. We apply this concept for the evolution of the kinetochore machinery in Euglenozoa, since no clear homology has so far been established among proteins constituting the conventional and kinetoplastid-specific kinetochores, except for the similarity between Ndc80/Nuf2 and KKIP1 in their disordered coil–coil regions. Moreover, our findings suggest that diplonemids and euglenids might possess the cenH3 variant. On the other hand, a novel BRCT-containing KKT4 protein serves as a microtubule-binding component in kinetoplastid kinetochores, a situation unique for eukaryotes. Indeed, some kinetoplastid kinetochore components appear to be recruited in a genus-specific manner (e.g., KKIPs 4 and 5 in *T. brucei*). Gene duplications and divergence are important drivers of the evolution of protein complexes [175], and once full genome sequences are available for a wider group of organisms, it will be interesting to assess their impact in Euglenozoa. In any case, we remain fully aware of the limitations imposed by the high rates of kinetochore as well as other proteins’ evolution onto the application of a range of bioinformatic methods for their identification. Another caveat is that many of these proteins are characterized by stage-specific expression and, thus, might be absent from conventional transcriptome assemblies. High-quality genome assemblies and experimental confirmations will be crucial for validating the results of our bioinformatics analyses.

## Conclusions

The Euglenozoa encompasses not just a large number of taxa, but a diverse collection of biological characters, the origin and evolution of which has proved puzzling as more and more unique processes and pathways have been described. A comprehensive reconstruction of these processes has not been possible due to the absence of both well-resolved trees and genome-wide data from diverse representative taxa. Having these in hand now allowed a deeper insight into the metabolism and processes shaping it and provided an opportunity to (re)analyze evolution of certain metabolic and molecular features, many of which were widely thought to be associated with the parasitic flagellates of the genera *Leishmania* and *Trypanosoma*. Our results suggest that trypanosomatids and bodonids as a whole, except for the free-living prokinetoplastids, are characterized by significantly lower metabolic capabilities compared to diplonemids, euglenids, and free-living heterotrophic protists in general. Gradual losses of genes encoding enzymes of amino acid, nucleotide, cofactor and vitamin metabolism, and other proteins occurred in both parasitic and free-living lineages and are not obviously tied to a major change in lifestyle, such as the origin of parasitism. Euglenids appear to possess more ancestral euglenozoan traits than other members of the phylum. These include a combination of trypanothione-, glutathione-, and thioredoxin-based systems in *Euglena*, as well as the least divergent pre-replication complex and kinetochore machinery. Diplonemids, on the contrary, are characterized by the presence of highly divergent (or unconventional) molecular machineries for chromosome segregation and DNA replication. Identification of ORC1, ORC2, and CDC6 in prokinetoplastids suggests that kinetoplastids pre-replication complex represents a highly divergent version of a classical eukaryotic machinery. The relationship between unconventional kinetoplastid and conventional eukaryotic kinetochore complexes is less clear and is even further complicated by the inability to identify clear homologues of either system in marine diplonemids.

## Materials and methods

### RNA isolation and transcriptome sequencing

Axenic cultures of *Rhynchopus humris* strain YPF1608 and *Sulcionema specki* strain YPF1618 were recently generated [18]. *Hemistasia phaeocysticola* strain YPF1303 was provided by Akinori Yabuki (JAMSTEC, Yokosuka, Japan). An axenic culture of *Trypanoplasma borreli* strain Tt-JH was isolated from a tench (*Tinca tinca*) [176] and kindly provided by Hanka Pecková (Institute of Parasitology). The RNA from three diplonemid species was isolated using Nucleospin RNA isolation kit (Macherey Nagel). The transcriptomic libraries of the diplonemids *H. phaeocysticola* (Hemistasiidae), *R. humris*, and *S. specki* (Diplonemidae) and the kinetoplastid *T. borreli* (Parabodonida) were prepared and sequenced on the Illumina HiSeq 4000 platform using the standard TruSeq protocol, resulting in ~ 53, ~ 124, ~ 106, and ~ 51 million paired-end unprocessed reads of 100 nt in length, respectively.

Clonal cultures of free-living eukaryovorous Prokinetoplatina strains PhM-4 and PhF-6 were isolated from brackish waters of Turkey and freshwaters of Vietnam, respectively. Total RNA was extracted using an RNAqueous-Micro Kit (Invitrogen, Cat. No. AM1931) and converted into cDNA using the Smart-Seq2 protocol [177]. Transcriptome sequencing was performed on the Illumina HiSeq 2500 platform with read lengths of 100 bp using the KAPA stranded RNA-seq kit (Roche) to construct paired-end libraries.

### Assembling the collection of transcriptomes and genomes

Transcriptomic reads of *H. phaeocysticola*, *R. humris*, *S. specki*, and *T. borreli* were subjected to adapter and quality trimming using Trimmomatic v.0.36 [178] with the following settings: maximal mismatch count, 2; palindrome clip threshold, 20; simple clip threshold, 10; minimal quality required to keep a base, 3; window size, 4; required quality, 15; and minimal length of reads to be kept, 75 nt. Transcriptome assemblies were generated using Trinity v.2.2.0 with minimal contig length set to 200 nt, with the “normalize_max_read_cov” option set to 50 for *R. humris*, and with the other parameters set at the default values [179].

Transcriptomic reads of PhM-4 and PhF-6 were quality trimmed with Trimmomatic-0.32 [178] with a maximum of two mismatches allowed, a sliding window size of 4 and minimum quality of 20, and a minimum length of 35. Trinity version 2.0.6 was used to assemble the dataset, using default values [179]. Transcriptome assembly steps were done in conjunction with an extensive prey sequence decontamination process (below).

The transcriptome libraries of *Rhabdomonas costata* strain PANT2 (Euglenida) were prepared from 4 μg of total RNA according to the standard TruSeq Stranded mRNA Sample Preparation Guide. Libraries were sequenced on an Illumina MiSeq instrument (Illumina, San Diego, CA, USA) using 150 base-length read chemistry in a paired-end mode. Reads were assembled by Trinity v2.0.6 into 93,852 contigs.

The assembled transcriptomes of *Neobodo designis* (Kinetoplastea, Neobodonida) and *Eutreptiella gymnastica* (Euglenida) were downloaded from the Marine Microbial Eukaryote Transcriptome Sequencing Project database (MMETSP) [11]. We used the transcriptome assembly of *Euglena gracilis* strain Z generated by Ebenezer et al. and that of *Azumiobodo hoyamushi* generated by Yazaki and colleagues [15, 180]. Redundant transcripts were filtered out from all the transcriptome assemblies using the CD-HIT-EST software v.4.6.7 [181] with the sequence identity threshold of 90%. Prediction of coding regions within transcripts was performed using Transdecoder v.3.0.0 [182] under the default settings, and the resulting files with protein sequences were used for further analyses. Completeness of the transcriptome and genome assemblies was assessed using the BUSCO v.3 software [53] and the “eukaryota_obd9” database containing a set of 303 universal eukaryotic single-copy orthologs.

Reference genome and transcriptome assemblies and sets of annotated proteins were downloaded from publicly available sources listed in Additional file 1: Table S1. For bodonids (i.e., Prokinetoplastina, Neo-, Para-, and Eubodonida), all genomes and transcriptomes publicly available at the time of the manuscript preparation were used. For trypanosomatids, five representative genome sequences were selected, two belonging to distantly related monoxenous (=one host) species (*P. confusum* and *L. pyrrhocoris*) and three to dixenous organisms (*T. brucei*, *T. grayi*, and *L. major*), switching between two hosts in their life cycles. Recently, *T. grayi* from crocodiles and *P. confusum* parasitizing mosquitoes were demonstrated to be slowly evolving trypanosomatids, preserving the highest number of ancestral genes [48]. *L. major* and *L. pyrrhocoris*, belonging to the subfamily Leishmaniinae, are characterized by different lifestyles [183]. *T. brucei* and *L. major* belong among the most extensively studied trypanosomatids and have high-quality genome assemblies and annotations available. The latter is also true for *L. pyrrhocoris* [51]

### Decontamination of the *R. costata*, *N. designis*, and Prokinetoplastina spp. transcriptomes

The culture of *R. costata* was non-axenic, and accordingly, the presence of transcripts belonging to contaminating species was detected using a BLASTN search against the SILVA database with an *E* value cut-off of 10^−20^ [184]. The best-scoring contaminants represented β- and γ-proteobacterial small-subunit (SSU) rRNA sequences. The following decontamination procedure was applied in order to get rid of the bacterial sequences: (i) a BLASTX search against the NCBI nr database using *R. costata* transcripts as queries with an *E* value cut-off of 10^−20^; (ii) the BLAST results were sorted according to the bitscore and only 20 best hits were retained for each *R. costata* query sequence; (iii) the best-scoring hits were annotated as “bacterial”, “eukaryotic”, and “other”; (iv) transcript sequences were considered to be of bacterial origin and excluded from further analyses if more than 60% of best hits were bacterial according to the results of classification at the previous step. The decontamination procedure described above and prediction of coding regions within the transcripts of non-bacterial origin has produced a dataset of 36,019 protein sequences, with 3679 proteins removed as bacterial contaminants.

A BLASTN search against the SILVA database using *N. designis* transcripts as queries with an *E* value cut-off of 10^−20^ revealed the presence of SSU rRNA sequences belonging only to a γ-proteobacterium of the genus *Alteromonas*. Since no other contaminants were identified, we downloaded all available genomes of *Alteromonas* spp. from the NCBI database and used them as a database for filtering out putative bacterial sequences from the *N. designis* transcriptome using BLASTN with an *E* value cut-off of 10^−5^. The contamination level was low, and this procedure resulted in removal of just 22 putative bacterial contigs from the transcriptome assembly.

As PhM-4 and PhF-6 are grown with the bodonids *Procryptobia sorokini*, and *Parabodo caudatus* as prey, respectively, we minimized contamination of the PhM-4 and PhF-6 datasets through an extensive bioinformatic decontamination procedure. This includes decontamination steps that took place before and after assembly of the PhM-4 and PhF-6 datasets. Before assembly of PhM-4 and PhF-6, we assembled 2 × 300 bp PE transcriptome reads from monoeukaryotic *P. sorokini* and *P. caudatus* prey cultures, along with 100 bp PE HiSeq 2000/2500 datasets derived from previously published datasets [185] in which other species preyed upon either *P. sorokini* or *P. caudatus* (i.e., cultures that were heavily contaminated by the same prey species). RNA-seq reads from PhM-4 and PhF-6 datasets were mapped to the assemblies containing *P. sorokini* or *P. caudatus* contigs, respectively, using Bowtie2 version 2.1.0 [186]. Reads that mapped to the prey assemblies (along with their mates, if only one read mapped) were discarded. The resulting unmapped reads were used to generate crude PhF-6 and PhM-4 transcriptome assemblies. To identify further prey-derived contamination, we used crude PhF-6 and PhM-4 assemblies to query the assembled transcriptomes of either *P. caudatus* or *P. sorokini* via megablast version 2.2.30 [187]. We considered a contig as a putative contaminant if it was ≥ 95% identical to sequences in the prey assemblies over a span of at least 75 bp. In the case of PhF-6, which was more extensively contaminated by prey than PhM-4, we added an additional step of mapping raw Illumina HiSeq2000 and MiSeq reads containing *P. caudatus* to the PhF-6 assembly; contigs with mapped reads were discarded. Potential cross-contamination from species multiplexed on the same HiSeq 2500 run was removed using the decontaminate.sh script from the BBMap package [188], with the options minc = 3, minp = 20, minr = 15, and minl = 350.

### Gene family inference and phylogenetic tree construction

Orthologous groups (OGs) containing proteins from 19 species (Additional file 1: Table S1) were inferred using OrthoFinder v.1.1.8 [189] under default settings. The heterolobosean *Naegleria gruberi* was used as an outgroup. For phylogenetic tree construction, OGs containing only one protein in each species were analyzed (52 OGs in total). Protein sequences of *R. costata* were additionally compared against the NCBI nr database with a relaxed *E* value cut-off of 10^−10^ in order to exclude any sequences of potential bacterial origin, which were not filtered out as described in the previous section with a more stringent *E* value cut-off of 10^−20^, but no contaminating sequences were identified. Inferred amino acid sequences of each gene were aligned using the L-INS-i algorithm in MAFFT v.7.310 [190]. The average percent identity within each OG was calculated using the alistat script from the HMMER package v.3.1 [77]. Twenty OGs demonstrating average percent identity within the group of > 50% were used for the phylogenomic analysis. The percent identity threshold was applied since our previous experience with euglenozoan phylogenomics [51, 191] shows that excluding highly divergent sequences improves the resolution of both maximum-likelihood and Bayesian trees. The protein alignments were trimmed using Gblocks v.0.91b with relaxed parameters (-b3 = 8, -b4 = 2, -b5 = h) and then concatenated, producing an alignment containing 6371 characters. A maximum-likelihood tree was inferred using IQ-TREE v.1.5.3 with the LG+F+I+G4 model and 1000 bootstrap replicates [192, 193]. A Bayesian phylogenetic tree was constructed using PhyloBayes-MPI v.1.7b [194] under the GTR-CAT model with four discrete gamma categories. Four independent Markov Chain Monte Carlo chains were run for ~ 8000 cycles, and all chains converged on the topology shown in Fig. 1. The initial 20% of cycles were discarded as a burn-in, and sampling every 5 cycles was used for inference of the final consensus tree visualized using FigTree v.1.4.3 [195].

### Analysis of metabolic pathways

For the analysis of metabolic capacities, an automatic assignment of KEGG Orthology (KO) identifiers to the proteins of the species of interest (Additional file 1: Table S1) was conducted using BlastKOALA v.2.1 [55]. The search was performed against a non-redundant pangenomic database of prokaryotes at the genus level and eukaryotes at the family level. KEGG Mapper v.2.8 was used for reconstruction of metabolic pathways and their comparison [196]. An enzyme was considered to be present in a particular group (diplonemids, euglenids, or kinetoplastids) if it was identified in at least two organisms belonging to that group (or in one species in the case of Prokinetoplastina). In certain cases, for verifying the original functional annotations, additional BLAST and/or Hidden Markov model-based (HMM) searches were performed with an *E* value cut-offs of 10^−20^ and 10^−5^, respectively, unless other parameters are specified. The number of metabolic proteins reported for a species is equal to the number of unique KO identifiers falling into the KEGG category “metabolism” assigned to the proteins encoded in the genome/transcriptome of that species. The term “metabolic proteins” is used herein to refer to the proteins belonging to the KEGG category “metabolism.” The analysis of protein sharing was performed using UpSetR package [197]. The unpaired *t* test was applied when necessary to test statistical significance of the observed differences in average number of unique KEGG identifiers across species groups.

For the comparison of metabolic capabilities of euglenozoans with those of other protists, high-quality genome assemblies of 16 free-living heterotrophic and 17 parasitic/symbiotic organisms were downloaded from the NCBI Genomes database (Additional file 1: Table S2). Assemblies demonstrating BUSCO coverage more than 75% for free-living species and 45% for parasites and symbionts were considered of high quality and analyzed using BlastKOALA v.2.1 as described for euglenozoans. A shared loss of a metabolic protein in kinetoplastids and ciliates was inferred if a protein was absent in both groups, while being present in at least three species of the free**-**living heterotrophic protists from other groups listed in Additional file 1: Table S2.

### Species clustering using the Uniform Manifold Approximation and Projection algorithm

Uniform Manifold Approximation and Projection (UMAP) is a novel general-purpose non-linear algorithm for dimensionality reduction [60]. The UMAP algorithm implemented in the uwot v0.1.3 R package [60] was applied to pairwise distances between 2181-dimensional vectors (presence/absence data for metabolic KO identifiers) for 19 species. First, we tried to find optimal values of key UMAP parameters that are suitable for recovering both local and global structure. The following setting combinations were tested: (1) the Euclidean or Hamming distance metrics, (2) number of nearest neighbors from 2 to 18, and (3) for each number of nearest neighbors, minimal distance between points in the 2D embedding was varied from 0 to 0.9 in 0.1 increments. The Euclidean and Hamming distance metrics yielded similar results, and the latter was selected as more appropriate for binary data. After inspecting all the resulting 2D embeddings, 3 was selected as the optimal number of nearest neighbors and 0 as the optimal minimal distance. Next, we ran 20 iterations of the algorithm with different random seeds generating both 2D and 3D embeddings of the multidimensional data structure. This was done to check whether the clustering remains stable across iterations. Results of 10 iterations are shown for both 2D (Additional file 6: Fig. S5) and 3D embeddings (Additional file 7: Fig. S6). The latter embeddings were visualized using the plot3D R package.

### Fatty acid biosynthesis

For the analysis of elongase repertoire, four proteins of *T. brucei* (TbELO1–4) described by Lee et al. [106] were used as a query in BLASTP search with an *E* value cut-off of 10^−20^ against the euglenozoan protein database. Phylogenetic trees were reconstructed using IQ-TREE with automatic model selection and 1000 bootstrap replicates for two datasets: (i) euglenozoan proteins only and (ii) euglenozoan sequences along with functionally characterized elongases from several other organisms (Additional file 14: File S1; Additional file 15: File S2) [109, 198–201]. For the identification of fatty acid synthase (FAS) I and II, proteins of *Saccharomyces cerevisiae* and *Homo sapiens* were used as queries with an *E* value cut-off of 10^−10^ [202, 203]. FAS I enzyme was considered to be present if at least three functional domains were identified on the same transcript.

### Analysis of trypanothione metabolism

Genes encoding the enzymes of the trypanothione biosynthetic pathway were considered to be present in a genome or transcriptome when the following conditions were fulfilled: (i) a protein could be identified by BLAST with an *E* value cut-off of 10^−20^ and/or a corresponding KEGG ID was assigned to a protein and (ii) p-distances between a reference protein and a putative hit calculated using MEGA v.7 did not exceed 0.7 or a different threshold specified in Additional file 13: Tables S41-S51 [204]. Additionally, the presence of a splice leader (SL) sequence was checked in the case of transcriptomic data, requiring a match with a minimal length of 12 nt. When a protein of interest could not be identified among predicted proteins, additional BLAST searches with raw transcriptome/genome sequences as a database were performed using an *E* value threshold of 10^−10^. For glutathionylspermidine (GspS) and trypanothione synthetases (TryS), as well as trypanothione (TR), glutathione (GR), and thioredoxin (TrxR) reductases, HMM-based searches using the HMMER package v.3.1 [77] were performed in addition to BLAST searches. An HMM model for GspS was generated using the Pfam seed alignment PF03738, and HMM models for other enzymes were obtained based on alignments of annotated sequences from the KEGG database. Two groups of proteins, GspS + TryS and TR represent related proteins, share a certain degree of sequence similarity and could be aligned (Additional file 13: Tables S50 and S51). For the identification of GspS/TryS homologues outside Euglenozoa, TryS of *T. brucei* was used as a query in a BLASTP search against the NCBI nr database (*E* value 10^−20^) and 1000 best hits for two groups, prokaryotes (group I) and other organisms (excluding Euglenozoa; group II), were obtained and combined into one file. Then, the sequences were filtered using CD-HIT-EST software v.4.6.7 [181] with 98% protein identity threshold. For the TR/GR/TrxR phylogeny, the corresponding protein sequences of *Emiliania huxleyi*, *Homo sapiens*, and trypanosomatids *Blechomonas ayalai*, *Endotrypanum monterogeii*, and *T. cruzi* were used as a reference. Sequences were aligned using Muscle v.3.8.31 with default parameters [205]. The resulting alignments were trimmed using trimAl v.1.4.rev22 with the “-strict” option [206]. Maximum-likelihood trees for both protein groups were build using IQ-TREE v.1.5.3 with 1000 and 100 bootstrap replicates, for reductases and synthases, respectively and the LG+I+G4 model (automatically selected). Bayesian trees were inferred using MrBayes v.3.2.6 with the models of rate heterogeneity across sites chosen based on IQ-TREE results, while models of amino acid substitutions were assessed during the analysis (mixed amino acid model prior). The resulting model was WAG+I+G4 for both synthetases and reductases. The analysis was run for one million generations with sampling every 100th of them and discarding the first 25% of samples as a burn-in.

### Identification of the DNA pre-replication complex subunits

Identification of the pre-replication complex (pre-RC) complex subunits was a multi-step procedure. Initially, BLAST searches with the reference sequences listed in Additional file 16: Table S52 as queries and an *E* value threshold of 10^−5^ against databases of annotated transcripts/genomes of the euglenozoans and protists belonging to other groups (Additional file 1: Table S2) were performed. If a target protein could not be identified, an HMM-based method was employed. Pre-computed models for the proteins of interest were downloaded from the Pfam database when available (Additional file 16: Table S52), or a new model was generated based on a protein alignment constructed using Muscle v.3.8.31 [205, 207]. When none or just a few euglenozoan proteins were identified, another round of HMM-based searches was performed. For that purpose, full-length reference sequences present in the seed alignment were downloaded from the Pfam database, and, when possible, high-scoring hits in euglenozoans and reference protists were added to the seed alignment (*E* value < 1^−20^_,_ preferably only full-length sequences with predicted domains). For HMM model construction, both trimmed and untrimmed alignments were used, and the search results were compared. Alignment trimming was accomplished in trimAl v.1.4.rev22 with the “-gappyout” option [206]. Visual inspection of phylogenetic trees constructed using IQ-TREE with automatic model selection and 1000 fast bootstrap replicates was performed to facilitate annotation of related sequences [192, 193].

Maximum-likelihood and Bayesian trees for the minichromosome maintenance (MCM) complex subunits 2–9 were inferred as described for the TR/GR/TrxR proteins, with the LG+F+I+G4 and WAG+I+G4 models, respectively. Only BLAST hits with p-distances ≤ 0.75 were considered. The trees were rooted using archaeal MCM sequences belonging to *Haloferax volcanii* (ADE04992), *Methanoculleus* sp. MAB1 (CVK32523.1), *Nanoarchaeum equitans* (NP_963571.1), and *Sulfolobus acidocaldarius* (WP_011277765.1).

Putative homologues of the winged-helix initiator protein were searched using an HMM model build based on an alignment of 35 archaeal sequences downloaded from the NCBI Protein database.

### Analysis of putative lateral gene transfer (LGT) events

For the analysis of putative LGT events, the protein sequences encoded by the genes of interest were used as a query in a BLASTP search against the NCBI nr database (*E* value 10^−20^) and 1000 best hits for each, prokaryotes and other organisms (excluding Euglenozoa), were obtained. The resulting sequences were filtered using CD-HIT-EST software v.4.6.7 [181] with 90–98% protein identity threshold (depending on the protein identity levels). Sequences were aligned using Muscle v.3.8.31 with default parameters [205], and the resulting alignment was trimmed with trimAl v.1.4.rev22 [206] and used for phylogenetic analyses. Maximum-likelihood and Bayesian trees were inferred as described for trypanothione biosynthetic enzymes with the automatically selected LG+I+G4 model and 100 standard bootstrap replicates (for maximum-likelihood analysis). The trees were visualized in FigTree v.1.4.3 [195].

### Identification of the kinetochore machinery elements

For the identification of putative centromeric histones H3 (cenH3), all available sequences of the canonical histone H3 (caH3) and its variants were downloaded from HistoneDB v.2.0 [208] and used as a BLAST query against transcripts, genomes, and predicted proteins of Euglenozoa with an *E* value threshold of 10^−5^. A hit was considered as a cenH3 candidate if it satisfied the following criteria: (i) at least one amino acid insertion in the loop 1 of the histone fold domain, (ii) divergent N-terminal tail, (iii) absence of the conserved glutamine residue in the α1 helix of the histone fold domain, and (iv) presence of a divergent histone fold domain [160]. Trypanosomatid-specific histone H3 variant (H3V) sequences were identified based on the presence of all of the following features: (i) a divergent N-terminal tail, (ii) absence of the conserved glutamine residue in the α1 helix of the histone fold domain, and (iii) absence of insertions in the loop 1 of the histone fold domain [209]. Distinguishing between putative caH3 and replication-independent histone variant H3.3, differing by only a few amino acids in opisthokonts [210], was out of scope of the current study, and the corresponding sequences were annotated as caH3/H3.3 (Additional file 9: Table S40).

Pre-computed HMMs for other conventional kinetochore components with the IDs specified in Additional file 16: Table S53 were downloaded from the Pfam database, and several rounds of HMM-based searches were performed as described for the DNA pre-replication complex subunits. Additionally, sequences of conventional kinetochore proteins identified by van Hooff and colleagues [158] in multiple eukaryotic lineages were used for building new HMMs, thus overcoming the bias towards overrepresentation of opisthokont sequences in the Pfam database. Only the most conserved components of the conventional kinetochore machinery were considered in our analyses, including the Ndc80 complex (Ndc80, Nuf2, Spc24, and Spc25 subunits), Knl1, the Mis12 complex (Mis12, Nnf1, Dsn1, and Nsl1), and CenpC.

For the identification of the kinetoplastid kinetochore proteins (KKTs), sequences annotated as KKTs were downloaded from the TriTryp database release 41, combined with the homologues identified in the eubodonid *Bodo saltans* [38], aligned using Muscle v.3.8.31 with default parameters [205], and used for HMM building and subsequent searches. Hits were annotated as putative KKTs when they met all of the following criteria: (i) HMM hit *E* value ≤ 10^−5^, (ii) p-distances calculated using MEGA v.7 did not exceed 0.8 or a different threshold specified in Additional file 13: Tables S13-S31 [204], and (iii) hit coordinates extending beyond predicted borders of highly conserved domains known to be present in proteins with unrelated functions. In the case of KKT2, 3, 10, and 19, HMM-based searches returned many hits due to the presence of widespread kinase domains [38, 162], and in order to facilitate annotation process, only two best hits for each species were taken for phylogenetic tree inference in IQ-TREE v.1.5.3 with 1000 fast bootstrap replicates (Additional file 17: File S3; Additional file 18: File S4; Additional file 19: File S5). Distinguishing between KKT10 and KKT19 proved to be a complicated task due to a very high degree of sequence similarity, and therefore, tentative annotation was performed based on the p-distances to the corresponding sequences in *B. saltans*.

Kinetoplastid kinetochore-interacting proteins (KKIPs) of *T. brucei* [163] were used as a BLAST query against the TriTryp database release 41 with an *E* value threshold of 10^−20^. Retrieved sequences were aligned and p-distances were calculated as described above. Hits with p-distances ≤ 0.8 to the homologues in *T. brucei* were aligned and used for HMM-based searches. The hits were filtered as described for the KKT proteins. For the phosphatase domain-containing KKIP7, only the hits with an *E* value ≤ 10^−100^ and p-distances ≤ 0.65 to the reference trypanosomatid sequences (Additional file 13: Tables S32-S38) were subjected to the phylogenetic analysis using IQ-TREE v.1.5.3 with 1000 fast bootstrap replicates (Additional file 20: File S6).

## Supplementary information

**Additional file 1: Table S1.** Genomes and transcriptomes of diplonemid, euglenid and kinetoplstid species analyzed in this study. *Naegleria gruberi* was used as an outgroup in phylogenomic analysis. **Table S2.** Genomes and transcriptomes of free-living and parasitic/symbiotic protists used in this study.

**Additional file 2: Figure S1.** Boxplots showing the number of metabolic proteins encoded in the genomes and transcriptomes of diplonemids, euglenids and kinetoplastids (panel A). The bottom and top of the box represent the first and third quartiles, respectively; the band inside the box corresponds to the median value. The length of the whiskers equals 1.5 * interquartile range. In panel B, the same species are grouped by their lifestyle: free-living or parasitic/symbiotic. The counts shown represent the number of unique KEGG identifiers from the category “metabolism” assigned to the annotated proteins of each analyzed species. Species abbreviations are as follows: Ahoya, *Azumiobodo hoyamushi*; Bsal, *Bodo saltans*; Egra, *Euglena gracilis*; Egym, *Eutreptiella gymnastica*; Hpha, *Hemistasia phaeocysticola*; Lmaj, *Leishmania major*; Lpyr, *Leptomonas pyrrhocoris*; Ndes, *Neobodo designis*; Pcon, *Paratrypanosoma confusum*; Perk, *Perkinsela* sp.; PhF-6, Prokinetoplastina sp. PhF-6; PhM-4, Prokinetoplastina sp. PhM-4; Rhum, *Rhynchopus humris*; Rcos, *Rhabdomonas costata*; Sspe, *Sulcionema specki*; Tbor, *Trypanoplasma borreli*; Tbru, *Trypanosoma brucei*; Tgra, *Trypanosoma grayi*.

**Additional file 3: Figure S2.** An UpSetR plot showing sharing of KEGG IDs assigned to metabolic proteins encoded in the genomes and transcriptomes of euglenids, diplonemids, free-living prokinetoplastids and other kinetoplastids. Pie charts contain annotations for ten most abundant KEGG functional categories exclusively shared among diplonemids and euglenids, as well as diplonemid- and kinetoplastid-specific ones and those exclusively shared among diplonemids, euglenids and free-living prokinetoplastids. A protein was considered present in a particular group if it was identified in at least one species belonging to the group.

**Additional file 4: Figure S3.** Boxplots showing the number of metabolic proteins encoded in the transcriptomes of free-living kinetoplastids, selected trypanosomatids and protists from several other groups. The counts shown represent the number of unique KEGG identifiers from the category “metabolism” assigned to the annotated proteins of each analyzed species. Species abbreviations are as follows: Bsal, *Bodo saltans*; Ndes, *Neobodo designis*; PhF-6, Prokinetoplastina sp. PhF-6; PhM-4, Prokinetoplastina sp. PhM-4. Data points for free-living kinetoplastids and ciliates are highlighted in yellow and cyan, respectively.

**Additional file 5: Figure S4.** The distribution of the KEGG identifiers belonging to the category “metabolism” absent in both, free-living kinetoplastids and ciliates, while being present in at least three species of free-living heterotrophic protists from other groups.

**Additional file 6: Figure S5.** Stability test of the UMAP ordination algorithm. We ran 20 iterations of the algorithm with different random seeds, with the number of nearest neighbors considered set to 3 and the minimal distance between samples in the embedding set to 0. Results for 10 iterations of 2D embedding are shown here. The clades are color-coded according to the legend, and the total number of unique KO identifiers per species is coded by point size. The following species abbreviations are used: A.hoy, *Azumiobodo hoyamushi*; B.sal, *Bodo saltans*; E.gym, *Eutreptiella gymnastica*; E.gra, *Euglena gracilis*; H.pha, *Hemistasia phaeocysticola*; L.maj, *Leishmania major*; L.pyr, *Leptomonas pyrrhocoris*; N.des, *Neobodo designis*; N.gru, *Naegleria gruberi*; P.con, *Paratrypanosoma confusum*; Perk, *Perkinsela* sp.; Phyl. F, prokinetoplastid species PhF-6; Phyl. M, prokinetoplastid species PhM-4; R.cos, *Rhabdomonas costata*; R.hum, *Rhynchopus humris*; Sulc., *Sulcionema specki*; T.bor, *Trypanoplasma borreli*; T.bru, *Trypanosoma brucei*; T.gra, *Trypanosoma grayi*.

**Additional file 7: Figure S6.** Stability test of the UMAP ordination algorithm. We ran 20 iterations of the algorithm with different random seeds, with the number of nearest neighbors considered set to 3 and the minimal distance between samples in the embedding set to 0. Results for 10 iterations of 3D embedding are shown here. Position of samples along the UMAP2 axis is color-coded.

**Additional file 8: Figure S7.** An UpSetR plot showing sharing of KEGG IDs assigned to metabolic proteins among six species clusters defined according to UMAP results (Fig. 2): 1/ Diplonemea; 2/ Euglenida (excluding *Rhabdomonas*); 3/ free-living prokinetoplastids; 4/ bodonids *Bodo saltans*, *Neobodo designis*, and *Azumiobodo hoyamushi*; 5/ the diverse cluster including *Naegleria gruberi*, *Trypanoplasma borreli*, *Paratrypanosoma confusum*, *Leishmania major*, and *Leptomonas pyrrhocoris*; 6/ *Trypanosoma brucei*, *Trypanosoma grayi*, and *Perkinsela* sp. Total counts of KEGG IDs in each species cluster (“set sizes”) are shown on the left. Few notable intersection sets are labeled in the figure.

**Additional file 9: Table S3.** Enzymes of essential amino acids biosynthesis. **Table S4.** Enzymes of methionine recycling. **Table S5.** Enzymes of amino acids degradation. **Table S6.** Enzymes of purine biosynthesis and salvage. **Table S7.** Enzymes of pyrimidine biosynthesis. **Table S8.** Enzymes involved in fatty acid biosynthesis in Euglenozoa. **Table S9.** Enzymes involved in the digestion of bacterial cell walls in Euglenozoa. **Table S10.** Euglenozoan enzymes involved in trypanothione biosynthesis and utulization. **Table S11.** Protein IDs of the components of the pre-replication complex in Euglenozoa. **Table S12.** Protein IDs for the elements of conventional and kinetoplastid-specific kinetochore machineries. **Table S40.** Centromeric and other variants of histone H3 identified in Euglenozoa.

**Additional file 10.** Euglenozoan proteins of amino acid metabolism, pre-replication complex and kinetochore.

**Additional file 11: Figure S8.** A phylogenetic tree of fumarate-dependent dihydroorotate dehydrogenases based on a trimmed alignment of 283 amino acids. Nodes exhibiting maximal bootstrap support and posterior probability (PP) are marked by black circles. Only bootstrap supports ≥50 and PP values ≥0.5 are shown. Clades of eukaryotic sequences are highlighted in yellow. Euglenozoan sequences analyzed in this study are shown on magenta background. **Figure S9.** A phylogenetic tree of D-lactate dehydrogenase sequences based on a trimmed alignment of 406 amino acids. Nodes exhibiting maximal bootstrap support and posterior probability (PP) are marked by black circles. Only bootstrap supports ≥50 and PP values ≥0.5 are shown. Clades of eukaryotic sequences are highlighted in yellow. Euglenozoan sequences analyzed in this study are shown on magenta background. **Figure S10.** A phylogenetic tree of inositol monophosphatase-like histidinol-phosphate phosphatases based on a trimmed alignment of 248 amino acids. Nodes exhibiting maximal bootstrap support and posterior probability (PP) are marked by black circles. Only bootstrap supports ≥50 and PP values ≥0.5 are shown. Clades of eukaryotic sequences are highlighted in yellow. Euglenozoan sequences analyzed in this study are shown on magenta background. **Figure S11.** A phylogenetic tree of histidinol-phosphate phosphatases belonging to the polymerase and histidinol-phosphate phosphatase protein family based on a trimmed alignment of 247 amino acids. Nodes exhibiting maximal bootstrap support and posterior probability (PP) are marked by black circles. Only bootstrap supports ≥50 and PP values ≥0.5 are shown. Clades of eukaryotic sequences are highlighted in yellow. Euglenozoan sequences analyzed in this study are shown on magenta background.

**Additional file 12: Figure S12.** Maps of pyrimidine and uracil degradation (panel A), and riboflavin, folate and thiamine biosynthesis (panels B, C and D, respectively) in diplonemids, euglenids and kinetoplastids. A protein is considered to be present in a group if it is identified in at least two species; for free-living prokinetoplastids and *B. saltans*/*N. designis* the presence of a gene is inferred if it is found in at least one species.

**Additional file 13: Tables S13-S51.** Estimates of evolutionary divergence among putative sequences of the following classes: KKTs (Table S13-S31), KKIPs (Tables S32-S38), centromeric histone H3 (Table S39) and enzymes of trypanothione biosynthesis and utilization (Tables S41-S51).

**Additional file 14: File S1.** A phylogenetic tree of elongases (euglenozoan proteins only). (TREEFILE 7 kb)

**Additional file 15: File S2.** A phylogenetic tree of elongases (euglenozoan sequences along with functionally characterized elongases from several other organisms). (TREEFILE 8 kb)

**Additional file 16: Table S52.** Protein IDs and Pfam database Hidden Markov model IDs used for the identification of pre-replication complex subunits. **Table S53.** Pfam database Hidden Markov model IDs used for identification of the elements of kinetochore machinery.

**Additional file 17: File S3.** A phylogenetic tree of euglenozoan KKT2 proteins. (TREEFILE 2 kb)

**Additional file 18: File S4.** A phylogenetic tree of euglenozoan KKT3 proteins. (TREEFILE 2 kb)

**Additional file 19: File S5.** A phylogenetic tree of KKT10 and KKT19 proteins. (TREEFILE 2 kb)

**Additional file 20: File S6.** A phylogenetic tree of euglenozoan KKIP7 proteins. (TREEFILE 3 kb)

## Data Availability

The datasets generated and analyzed during the current study are available at DDBJ/ENA/GenBank under the following BioProject accessions: PRJNA549599 [211] (*H. phaeocysticola*), PRJNA549754 [212] (Prokinetoplastina spp. PhF-6 and PhM-4), PRJNA549827 [213] (*T. borreli*), PRJNA550027 [214] (*S. specki* and *R. humris*), and PRJNA550357 [215] (*R. costata*). All other data generated or analyzed during this study are included in this article and its supplementary information files.
