# Supplementary figures and images for: Evolution of metabolic capabilities and molecular features of diplonemids, kinetoplastids, and euglenids
Source: BMC Biol. 2020 Mar 2;18:23. doi: 10.1186/s12915-020-0754-1 (PMC7052976; doi:10.1186/s12915-020-0754-1)

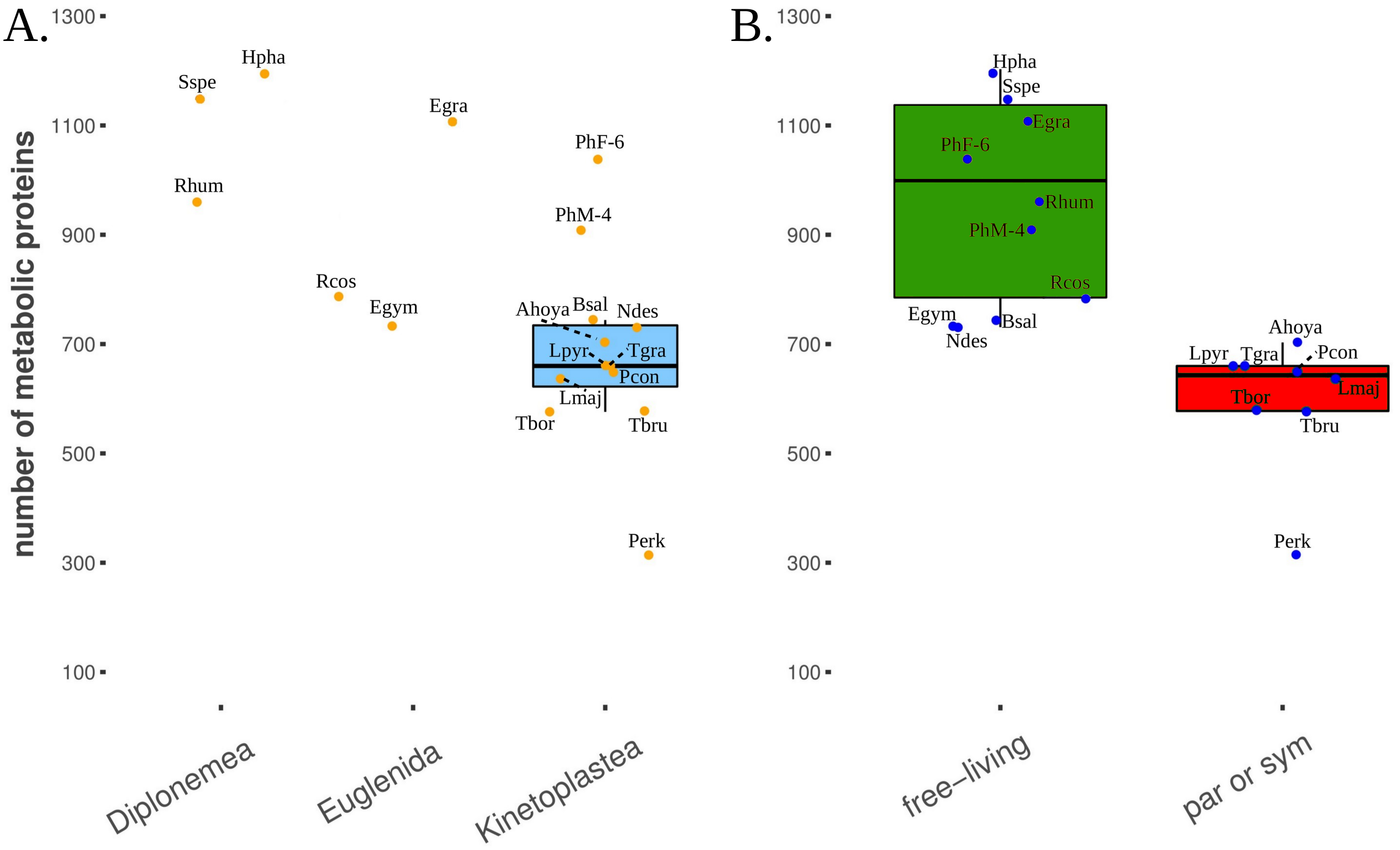

Supplement: Supplementary file 2 — Additional file 2: Figure S1. Boxplots showing the number of metabolic proteins encoded in the genomes and transcriptomes of diplonemids, euglenids and kinetoplastids (panel A). The bottom and top of the box represent the first and third quartiles, respectively; the band inside the box corresponds to the median value. The length of the whiskers equals 1.5 * interquartile range. In panel B, the same species are grouped by their lifestyle: free-living or parasitic/symbiotic. The counts shown represent the number of unique KEGG identifiers from the category “metabolism” assigned to the annotated proteins of each analyzed species. Species abbreviations are as follows: Ahoya, Azumiobodo hoyamushi; Bsal, Bodo saltans; Egra, Euglena gracilis; Egym, Eutreptiella gymnastica; Hpha, Hemistasia phaeocysticola; Lmaj, Leishmania major; Lpyr, Leptomonas pyrrhocoris; Ndes, Neobodo designis; Pcon, Paratrypanosoma confusum; Perk, Perkinsela sp.; PhF-6, Prokinetoplastina sp. PhF-6; PhM-4, Prokinetoplastina sp. PhM-4; Rhum, Rhynchopus humris; Rcos, Rhabdomonas costata; Sspe, Sulcionema specki; Tbor, Trypanoplasma borreli; Tbru, Trypanosoma brucei; Tgra, Trypanosoma grayi. [file 12915_2020_754_MOESM2_ESM.tif]

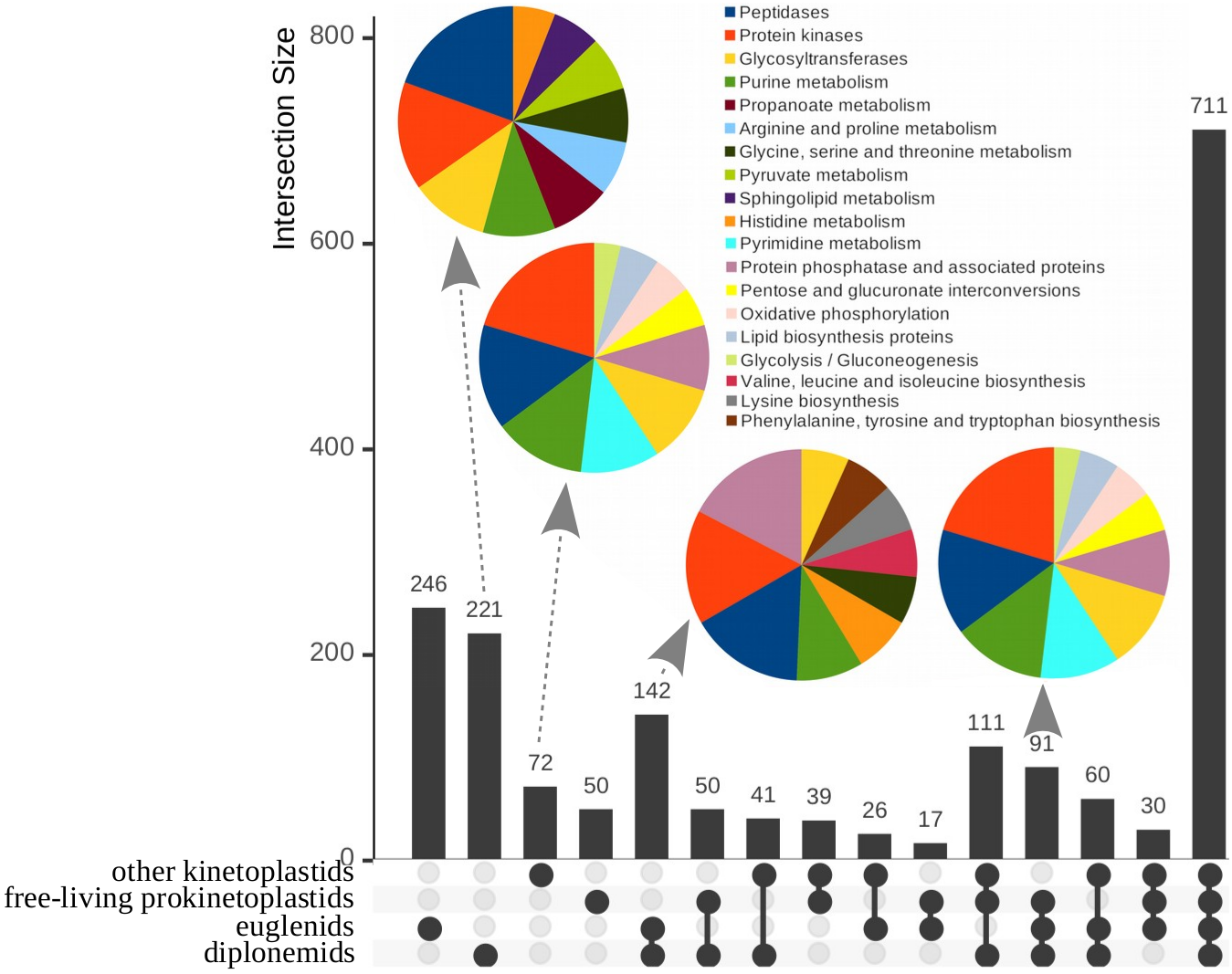

Supplement: Supplementary file 3 — Additional file 3: Figure S2. An UpSetR plot showing sharing of KEGG IDs assigned to metabolic proteins encoded in the genomes and transcriptomes of euglenids, diplonemids, free-living prokinetoplastids and other kinetoplastids. Pie charts contain annotations for ten most abundant KEGG functional categories exclusively shared among diplonemids and euglenids, as well as diplonemid- and kinetoplastid-specific ones and those exclusively shared among diplonemids, euglenids and free-living prokinetoplastids. A protein was considered present in a particular group if it was identified in at least one species belonging to the group. [file 12915_2020_754_MOESM3_ESM.tif]

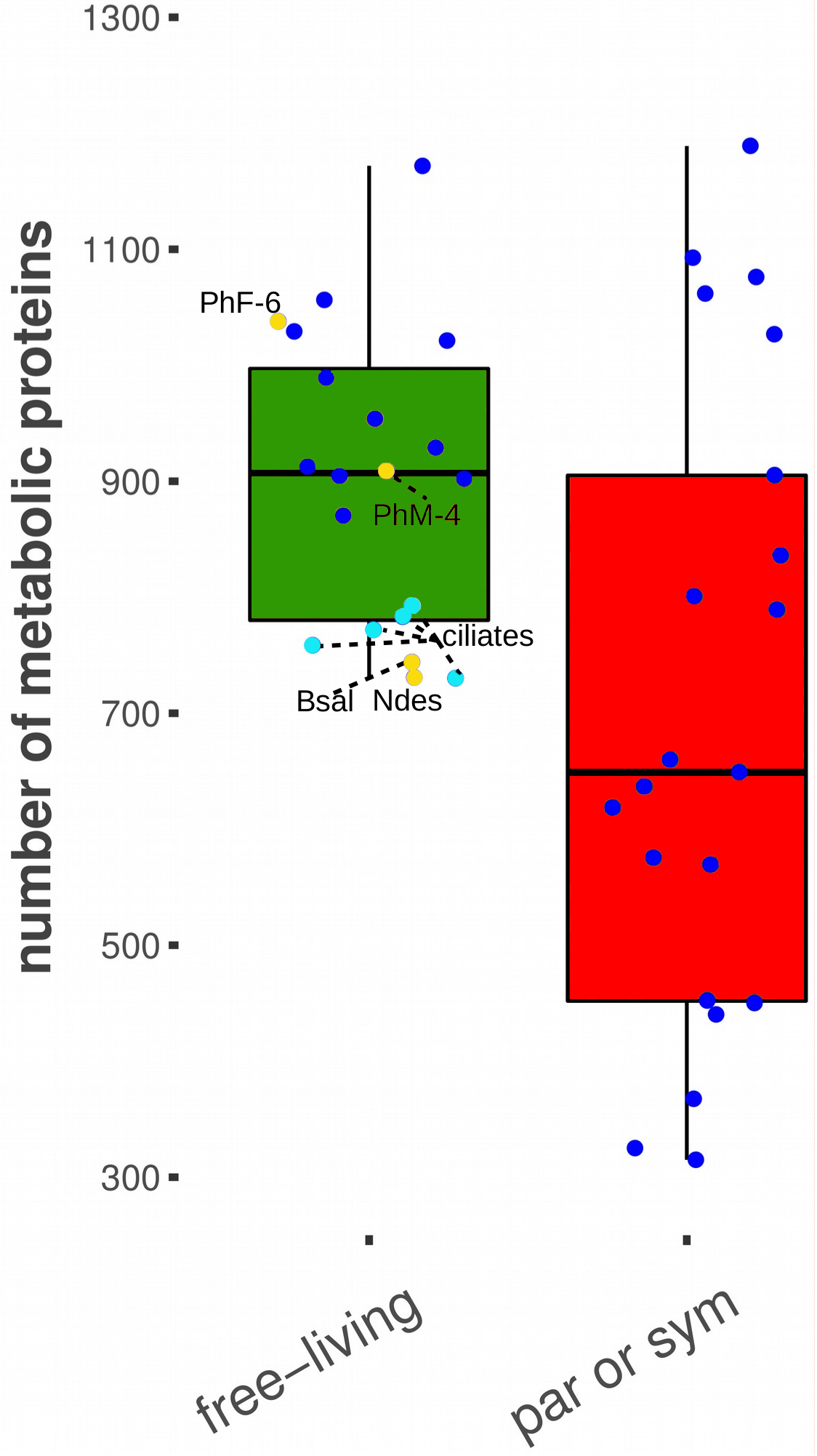

Supplement: Supplementary file 4 — Additional file 4: Figure S3. Boxplots showing the number of metabolic proteins encoded in the transcriptomes of free-living kinetoplastids, selected trypanosomatids and protists from several other groups. The counts shown represent the number of unique KEGG identifiers from the category “metabolism” assigned to the annotated proteins of each analyzed species. Species abbreviations are as follows: Bsal, Bodo saltans; Ndes, Neobodo designis; PhF-6, Prokinetoplastina sp. PhF-6; PhM-4, Prokinetoplastina sp. PhM-4. Data points for free-living kinetoplastids and ciliates are highlighted in yellow and cyan, respectively. [file 12915_2020_754_MOESM4_ESM.tif]

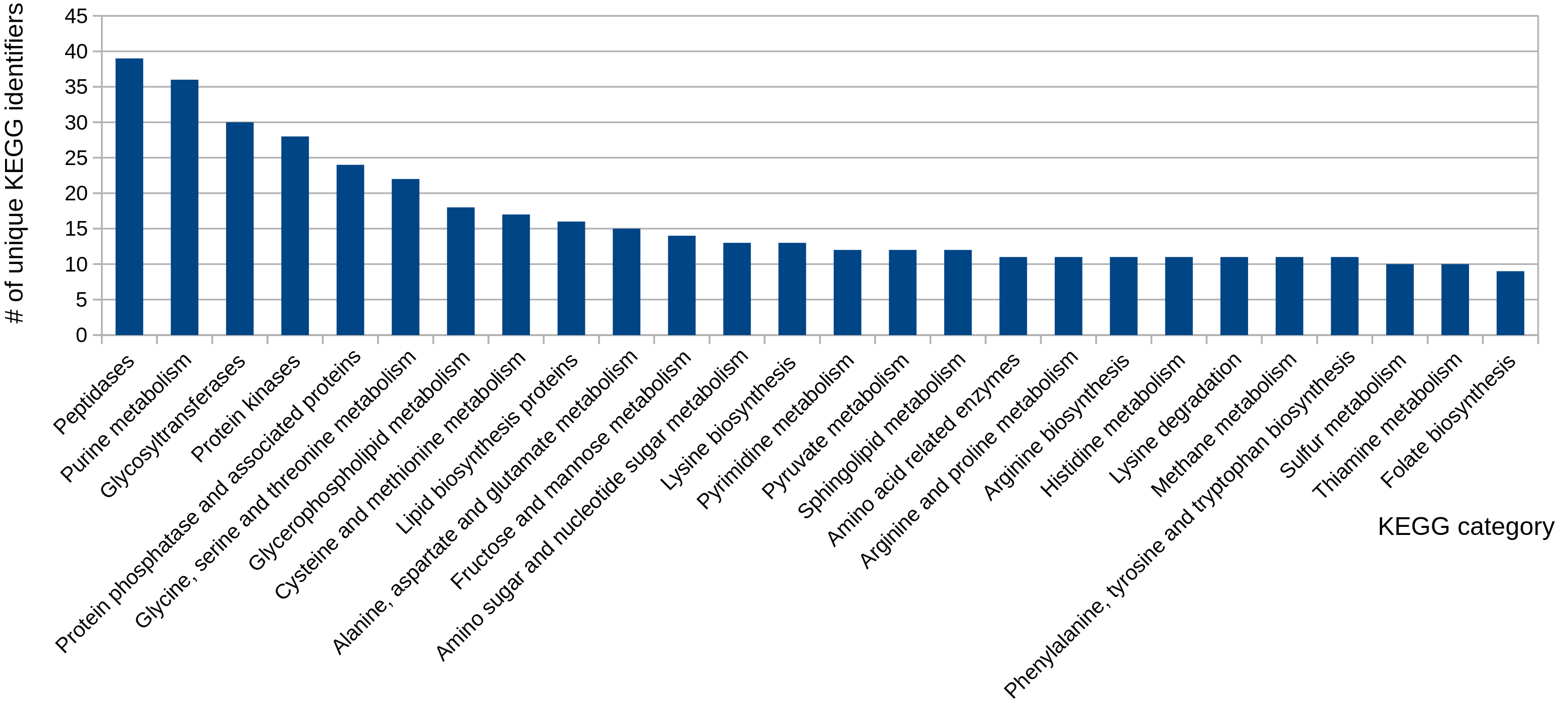

Supplement: Supplementary file 5 — Additional file 5: Figure S4. The distribution of the KEGG identifiers belonging to the category “metabolism” absent in both, free-living kinetoplastids and ciliates, while being present in at least three species of free-living heterotrophic protists from other groups. [file 12915_2020_754_MOESM5_ESM.tif]

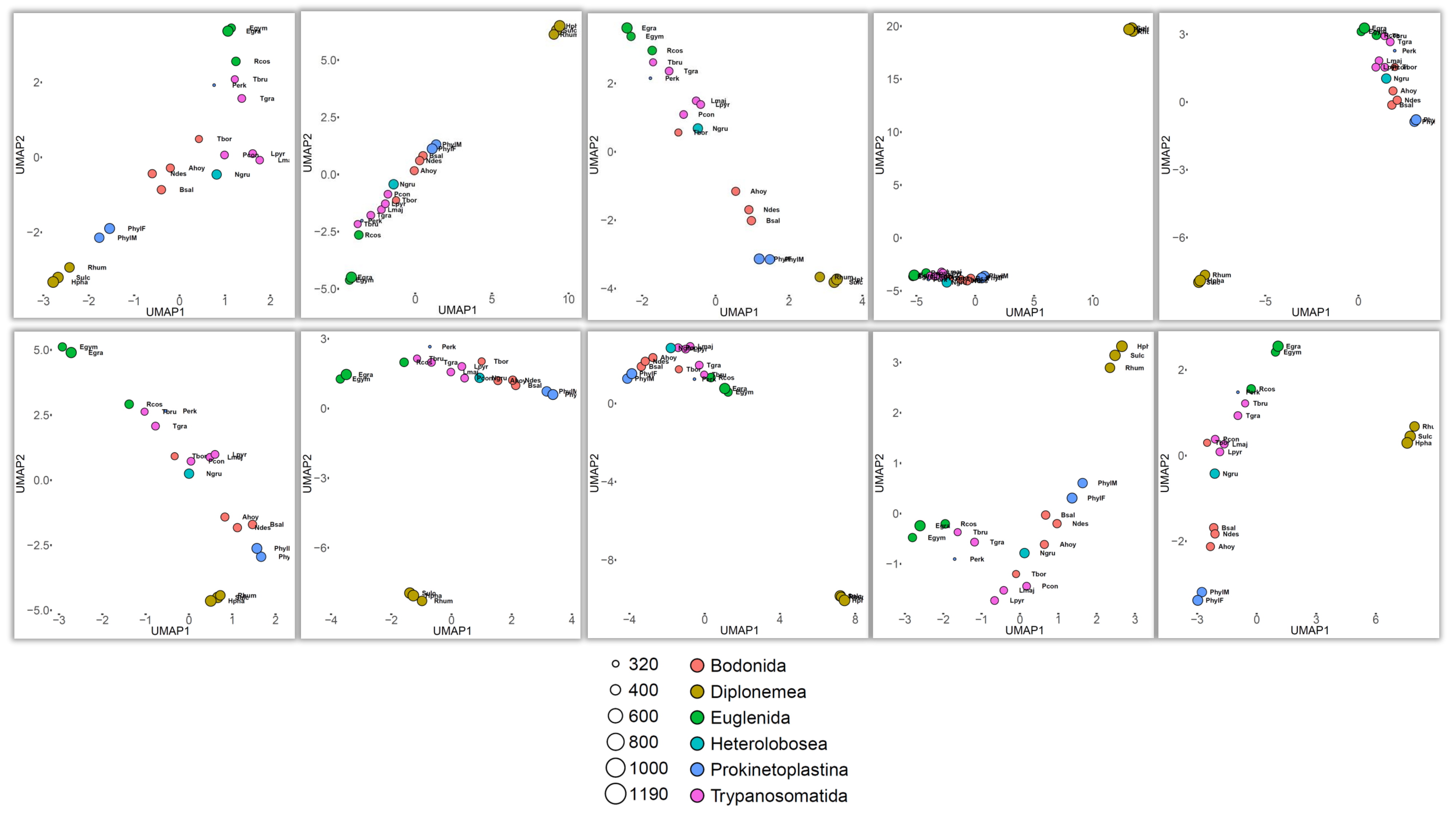

Supplement: Supplementary file 6 — Additional file 6: Figure S5. Stability test of the UMAP ordination algorithm. We ran 20 iterations of the algorithm with different random seeds, with the number of nearest neighbors considered set to 3 and the minimal distance between samples in the embedding set to 0. Results for 10 iterations of 2D embedding are shown here. The clades are color-coded according to the legend, and the total number of unique KO identifiers per species is coded by point size. The following species abbreviations are used: A.hoy, Azumiobodo hoyamushi; B.sal, Bodo saltans; E.gym, Eutreptiella gymnastica; E.gra, Euglena gracilis; H.pha, Hemistasia phaeocysticola; L.maj, Leishmania major; L.pyr, Leptomonas pyrrhocoris; N.des, Neobodo designis; N.gru, Naegleria gruberi; P.con, Paratrypanosoma confusum; Perk, Perkinsela sp.; Phyl. F, prokinetoplastid species PhF-6; Phyl. M, prokinetoplastid species PhM-4; R.cos, Rhabdomonas costata; R.hum, Rhynchopus humris; Sulc., Sulcionema specki; T.bor, Trypanoplasma borreli; T.bru, Trypanosoma brucei; T.gra, Trypanosoma grayi. [file 12915_2020_754_MOESM6_ESM.tif]

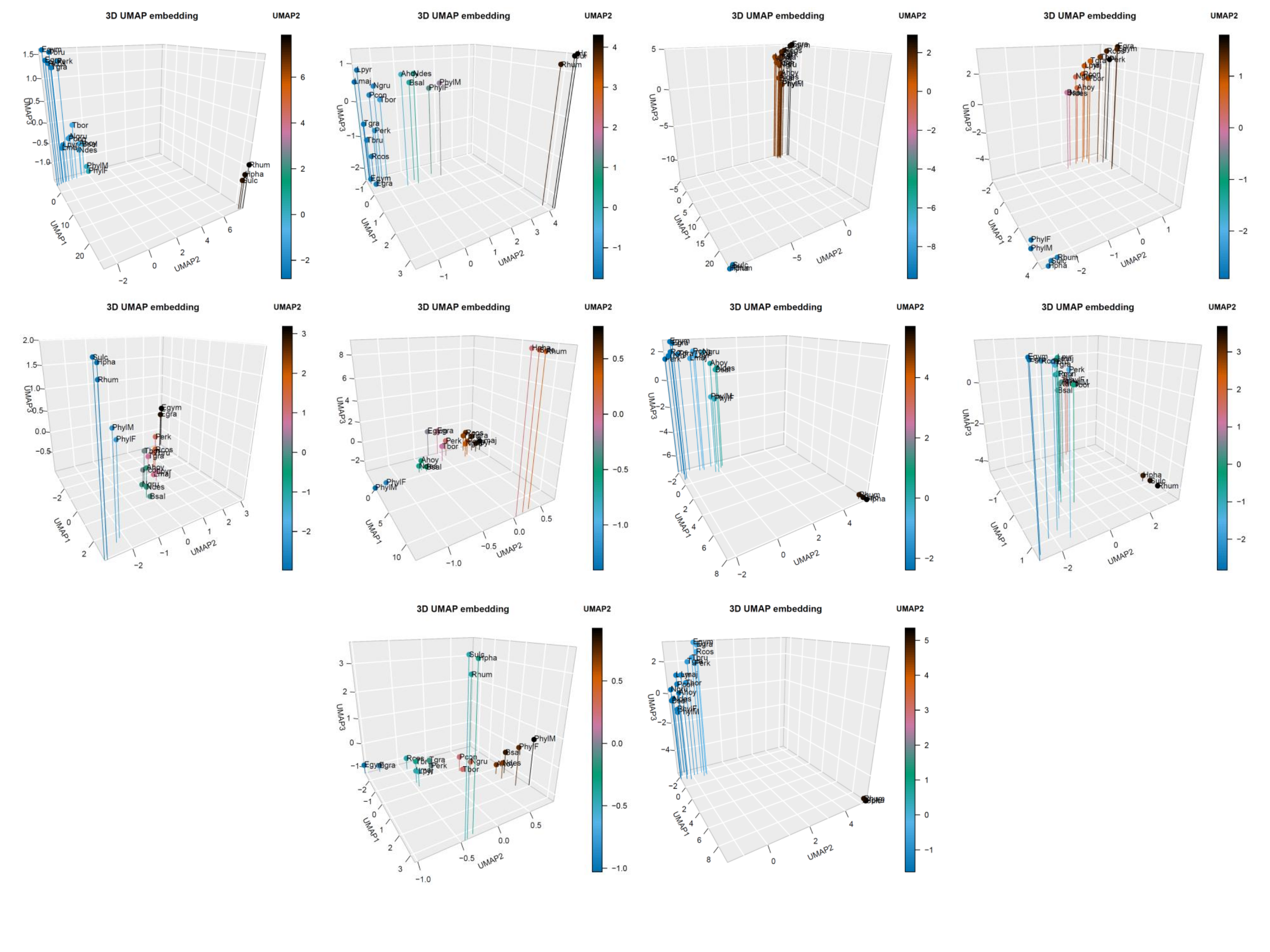

Supplement: Supplementary file 7 — Additional file 7: Figure S6. Stability test of the UMAP ordination algorithm. We ran 20 iterations of the algorithm with different random seeds, with the number of nearest neighbors considered set to 3 and the minimal distance between samples in the embedding set to 0. Results for 10 iterations of 3D embedding are shown here. Position of samples along the UMAP2 axis is color-coded. [file 12915_2020_754_MOESM7_ESM.tif]

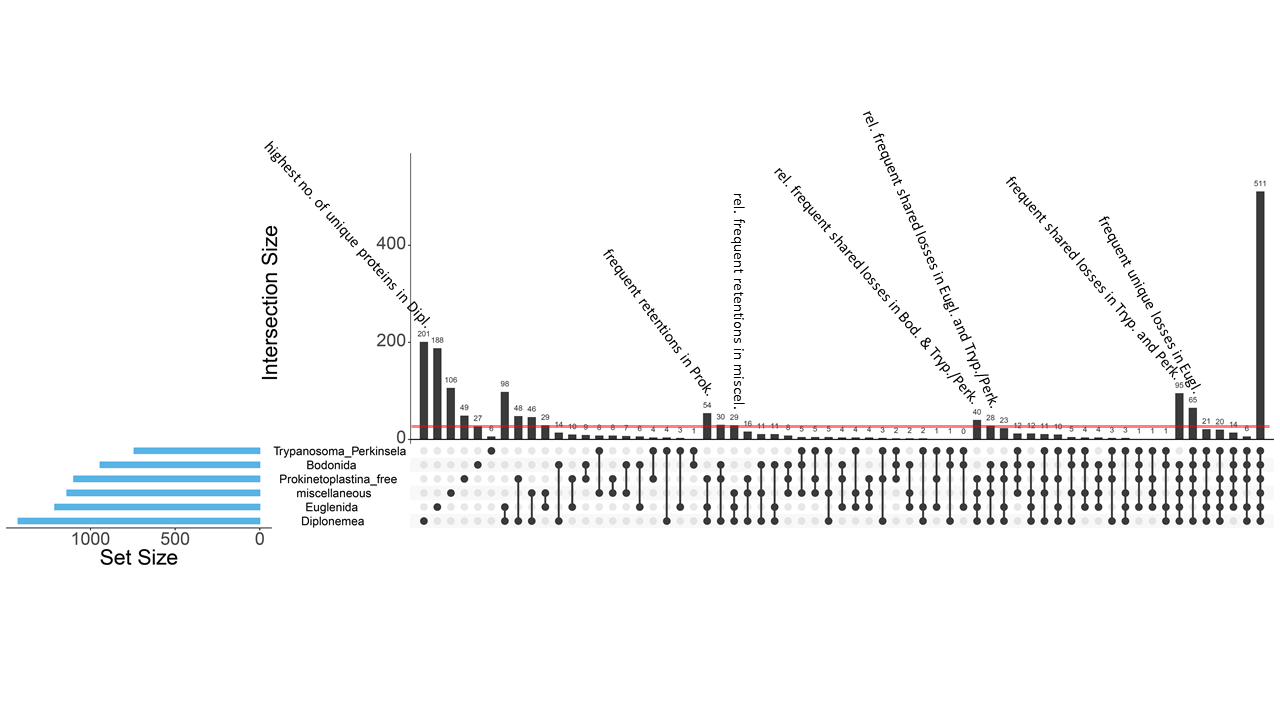

Supplement: Supplementary file 8 — Additional file 8: Figure S7. An UpSetR plot showing sharing of KEGG IDs assigned to metabolic proteins among six species clusters defined according to UMAP results (Fig. 2): 1/ Diplonemea; 2/ Euglenida (excluding Rhabdomonas); 3/ free-living prokinetoplastids; 4/ bodonids Bodo saltans, Neobodo designis, and Azumiobodo hoyamushi; 5/ the diverse cluster including Naegleria gruberi, Trypanoplasma borreli, Paratrypanosoma confusum, Leishmania major, and Leptomonas pyrrhocoris; 6/ Trypanosoma brucei, Trypanosoma grayi, and Perkinsela sp. Total counts of KEGG IDs in each species cluster (“set sizes”) are shown on the left. Few notable intersection sets are labeled in the figure. [file 12915_2020_754_MOESM8_ESM.tif]

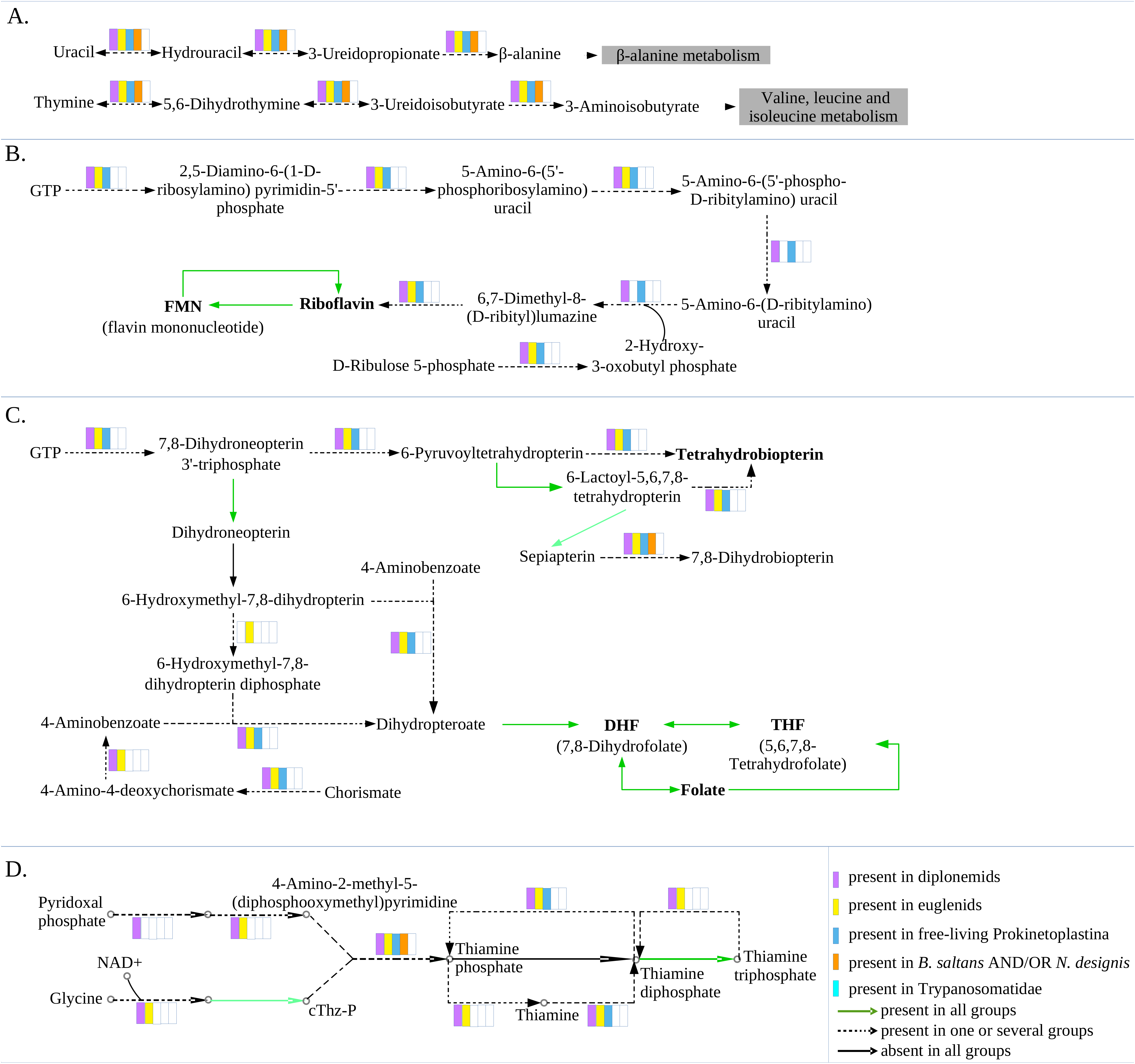

Supplement: Supplementary file 12 — Additional file 12: Figure S12. Maps of pyrimidine and uracil degradation (panel A), and riboflavin, folate and thiamine biosynthesis (panels B, C and D, respectively) in diplonemids, euglenids and kinetoplastids. A protein is considered to be present in a group if it is identified in at least two species; for free-living prokinetoplastids and B. saltans/N. designis the presence of a gene is inferred if it is found in at least one species. [file 12915_2020_754_MOESM12_ESM.tif]
