# Supplementary material for: Evolution of metabolic capabilities and molecular features of diplonemids, kinetoplastids, and euglenids
Source: BMC Biol. 2020 Mar 2;18:23. doi: 10.1186/s12915-020-0754-1 (PMC7052976; doi:10.1186/s12915-020-0754-1)
Supplement: Supplementary file 10 — Additional file 10. Euglenozoan proteins of amino acid metabolism, pre-replication complex and kinetochore. [file 12915_2020_754_MOESM10_ESM.docx]

**Euglenozoan proteins of amino acid metabolism, pre-replication complex and kinetochore**

**Amino acid biosynthesis**

Diplonemids and euglenids possess a much broader set of amino acid biosynthesis enzymes than kinetoplastids (Fig. 3; Additional file 9: Table S3). Both groups of protists share the ability to synthesize valine, leucine and isoleucine from pyruvate. Furthermore, all enzymes involved in histidine biosynthesis, except for histidinol-phosphate phosphatase (HPP), were identified in these organisms. HPP, catalyzing the penultimate step of histidine biosynthesis, was not identified using BlastKOALA, and therefore we performed an additional BLASTP search with an E-value cut-off of 10^-20^ and with bacterial, yeast and plant HPP sequences as queries. Using this strategy, we identified an HPP homolog in *E. gracilis* belonging to the family of inositol monophosphatase-like (IMP) proteins recently characterized in *Actinobacteria*, *Corynebacterium* spp. and plants [68, 69]. The protein from *E. gracilis* falls within a bacterial clade on the phylogenetic tree (Additional file 11: Figure S10) and, importantly, has all the characteristic sequence motifs of an IMP-like HPP [69]. The transcriptome of PhF-6 encodes HPP belonging to the polymerase and histidinol-phosphate phosphatase subfamily and clusters within a eukaryotic clade (Additional file 11: Figure S11). Diplonemids possibly possess divergent histidinol-phosphatases yet to be identified. Interestingly, the diplonemids *R. humris* and *S. specki* carry a homologue of the trifunctional His4 protein (phosphoribosyl-ATP pyrophosphohydrolase/phosphoribosyl-AMP cyclohydrolase/histidinol dehydrogenase), while *H. phaeocysticola* and euglenids possess separate transcripts.

In diplonemids, euglenids, and free-living prokinetoplastids phosphoenolpyruvate and erythrose 4-phosphate are converted to chorismate, an end product of the shikimate pathway and a precursor of the aromatic amino acids, folate and ubiquinone. Notably, steps 2 through 6 of the shikimate pathway in euglenozoans are catalyzed by a multi-functional AROM protein, previously found only in prokaryotes, fungi, apicomplexa, ciliates, and oomycetes, while the first and the last steps are catalyzed by 3-deoxy-7-phosphoheptulonate synthase and chorismate synthase, respectively [65–67]. The identified AROM sequences exhibit ~30% identity to the corresponding proteins from a ciliate *Tetrahymena thermophila* and an apicomplexan *Toxoplasma gondii* and share with them the following domain arrangement: 3-dehydroquinate synthase, 5-enolpyruvylshikimate-3-phosphate synthase, shikimate kinase, 3-dehydroquinate dehydratase I, and shikimate dehydrogenase*.*

Diplonemids and euglenids are likely capable of tryptophan biosynthesis. *E. gracilis* is known to synthesize tyrosine and phenylalanine from L-arogenate, similarly to plants and some bacteria, while most other organisms use the phenylpyruvate/4-hydroxyphenylpyruvate pathways [70, 71]. We have identified the arogenate/prephenate dehydratases and dehydrogenases in both diplonemids and euglenids. Although it is difficult to infer substrate specificity of arogenate/prephenate dehydratases and dehydrogenases using the protein sequence alone, we suppose that diplonemids synthesize tyrosine *via* hydroxyphenylpyruvate, and not arogenate, since they also possess the second enzyme, tyrosine aminotransferase, not identified in euglenids, which are likely to use the arogenate pathway. Notably, the *E. gracilis* protein appears to be bifunctional, carrying an arogenate/prephenate dehydratase domain at the N-terminus and a dehydrogenase at the C-terminus.

Some euglenozoans are capable of lysine biosynthesis from diaminopimelate (DAP) and *meso*-diaminopimelate found in bacterial cell walls [72, 73]. *N. designis*, PhF-6 and *L. pyrrhocoris* possess both enzymes of the pathway, DAP epimerase and DAP decarboxylase, while *R. costata*, *H. phaeocysticola*, *S. specki*, PhM-4, *A. hoyamushi* and *B. saltans* possess only the latter protein. Diplonemids and euglenids appear to be capable of lysine biosynthesis from 2-oxoglutarate *via* the α-aminoadipate pathway (Fig. 3). Two enzymes of the pathway, homoisocitrate dehydrogenase and saccharopine dehydrogenase (NAD+), were not identified in diplonemid and euglenid transcriptomes, respectively, and homoaconitase appears to be absent in both groups. However, the presence of the other enzymes of the pathway in the analyzed transcriptomes and reports of the α-aminoadipate pathway in *Euglena* [74] support the idea that it is functional in these protists.

**Composition of the DNA pre-replication complex (pre-RC)**

Four main components of the eukaryotic pre-RC (hexameric origin recognition (ORC) and minichromosome maintenance (MCM) complexes, proteins CDC6 and Cdt1) are known to exhibit widely variable levels of sequence conservation [75]. Therefore, for identification of these proteins we have employed BLAST along with several rounds of more sensitive HMM-based searches [76, 77].

The subunits of MCM complex (MCM2-7) were readily identified in almost all euglenozoan genomes and transcriptomes (Fig. 7; Additional file 9: Table S11). In addition, we identified MCM8 and MCM9 proteins, which are related to the MCM complex subunits and are involved in DNA replication in various eukaryotic groups [78, 79]. Although both proteins are slightly more divergent in kinetoplastids than in other euglenozoans, they could still be readily identified by BLAST using the sequences of *A. thaliana* and *Homo sapiens* as queries (Fig. 7; Additional file 9: Table S11).

MCM2 through 9 belong to the same protein family, share domains, and in agreement with previous studies, form 8 well-supported clades (Fig. 7) [80]. The tree topology suggests *MCM8* and *9* are products of gene duplication. Due to a very low resolution at the backbone of the maximum-likelihood tree, it is impossible to confidently establish whether *MCM2* and *3*, *4* and *7*, as well as *5* and *6* also emerged by gene duplication. While less conserved, MCM10, which is unrelated to the other MCM complex subunits [81, 82], could still be identified in all euglenozoans analyzed here except for *E. gymnastica*.

Another component of the eukaryotic pre-RC complex, the Cdt1 protein, could be identified only in the transcriptome of *E. gracilis*. The Cdt1 protein found in this study is 99% and 97% identical to the *E. gracilis* sequences from other strains, all of which are recognizable by Pfam database search [12, 13].Subunit 1 of the ORC complex and CDC6 protein, which binds to ORC1 and stabilizes the ORC on replication origins, are related and highly similar [83, 84]. We have used homology-based searches combined with a phylogenetic analysis to assign functions to the identified sequences. This approach enabled identification of putative homologues of both proteins in all diplonemids, euglenids and free-living Prokinetoplastina. Except for the latter clade, only one sequence bearing resemblance to both ORC1 and CDC6 could be identified in kinetoplastids. We refer to these proteins as ORC1/CDC6 (Fig. 7). Full-length ORC1 sequences of euglenozoans lack the bromo-adjacent-homology domain, usually found in the N-terminal regions of opisthokont ORC1, being responsible for recognition of specific histone modifications at the replication origins in metazoans and stabilizing ORC-origin interactions in yeast [85, 86]. Orthologues of ORC1b, representing a remote homologue of ORC1 subunit in trypanosomatids [87], were initially identified as weak hits to the ORC1 in *T. brucei*, *L. major*, *L. pyrrhocoris*, and *T. borreli*. Using these sequences as queries, we have found their orthologues in all trypanosomatids and bodonids within our dataset. In PhF-6, we could identify only a very short partial sequence, which cannot be unambiguously annotated as an ORC1b homologue.

ORC3 subunit of the pre-RC complex is characterized by the highest degree of divergence and could not be identified in any species within our dataset. Putative homologues of ORC5 were found only in euglenids, while ORC2 is present in *E. gracilis*, *E. gymnastica* and PhM-4. The latter sequence is 33% identical to its counterpart in *E. gracilis*, contains an ORC2 domain recognized using a Pfam database search and originates from a transcript bearing the SL sequence, thus excluding contamination. Orthologues of the ORC4 subunit are absent from diplonemids, *R*. *costata*, *A*. *hoyamushi*, and *T. borreli*.

The ORC6 proteins have no sequence similarity to the ORC1-5 subunits and CDC6 belonging to the AAA+ family and sharing the winged-helix DNA-binding domain [83]. In our study ORC6 homologues with recognizable ORC6 domains could be confidently identified only in euglenids, while in diplonemids putative hits do not possess any recognizable domains and their functional role remains to be elucidated experimentally.

**Kinetochore elements**

We have performed sensitive HMM-based searches of both conventional opisthokont (Ndc80, Mis12 and Knl1 complexes) and unconventional kinetoplastid kinetochore (20 KKT and 7 KKIP proteins) components in Euglenozoa.

The vast majority of KKT proteins were identified in trypanosomatids, with the exception of KKT4 and 19 in *T. grayi*, and KKT20 in *P. confusum* (Fig. 8; Additional file 9: Table S12). Only the putative homologues of KKT13, 10 and 19 were identified outside Kinetoplastea. Kinase domain-carrying sequences of KKT10 and 19 of *H. phaeocysticola* and *E. gracilis* demonstrate 64 and 46% identity, respectively. A BLAST search in the NCBI non-redundant protein (nr) database with putative euglenid and diplonemid KKT10 and 19 as queries yielded the respective trypanosomatid proteins as best hits. Euglenid KKT13 homologs exhibit 50-60% identity and 20-30% query coverage when aligned with a corresponding sequence of *B. saltans*. Interestingly, various bacterial FHA domain-carrying proteins appear as the best BLAST hits outside of Euglenozoa, requiring further investigation of the origin of genes encoding kinetochore proteins. HMM-based searches also returned a hit to KKT13 in *S. specki* with an E-value of 10^-8^. However, the hit was entirely restricted to the borders of the FHA domain predicted using InterProScan [88] and, thus, disregarded.

Among KKIP proteins, only the putative homologues of KKIP7 could be identified outside Kinetoplastea. KKIP1, 4 and 5 appear to be confined to trypanosomatids, while putative homologues of KKIP2, 3 and 6 were identified in bodonids and prokinetoplastids (the latter group possesses only KKIP2 and KKIP7). Moreover, KKIP4 and 5 might be specific for the genus *Trypanosoma*, since only a very divergent hit with a p-distance of ~0.7 to its counterpart in *T. brucei* is present in *P. confusum*, and no homologues were found in *Leptomonas* and *Leishmania* spp.

Based on several criteria (see Materials and Methods for details) we have identified putative centromeric histone H3 (cenH3) candidates in diplonemids (*H. phaeocysticola* and *R. humris*), euglenids (*E. gracilis* and *R. costata*), a neobodonid *N. designis*, and free-living prokinetoplastids. Although we have used several criteria for assigning putative cenH3, previous studies demonstrated that even the main bioinformatic criterion for its identification, namely the presence of a longer DNA-interacting loop 1 within the histone-fold domain, occasionally leads to misannotations. For example, the *Giardia intestinalis* genome bears two sequences satisfying the criteria for being a cenH3, and yet only one of them encodes *bona fide* cenH3, while cenH3 in *Dictyostelium discoideum* does not have an insertion in the loop 1 [89, 90]. Thus, experimental evidence is required in case of euglenozoan cenH3 candidates. The analysis of other euglenozoan H3 variants also led us to a conclusion that H3V, known to regulate RNA polymerase II transcription termination in trypanosomatids [91], is confined to Kinetoplastea.

The elements of the Mis12 kinetochore complex, consisting of Mis12, Nnf1, Dsn1, and Nsl1 in humans and yeast, and the Knl1 protein, forming a so-called KMN network together with the Mis12 and Ndc80 complexes could not be identified in any Euglenozoa [92, 93].
