# Supplementary material for: Evolution of metabolic capabilities and molecular features of diplonemids, kinetoplastids, and euglenids
Source: BMC Biol. 2020 Mar 2;18:23. doi: 10.1186/s12915-020-0754-1 (PMC7052976; doi:10.1186/s12915-020-0754-1)
Supplement: Supplementary file 11 — Additional file 11: Figure S8. A phylogenetic tree of fumarate-dependent dihydroorotate dehydrogenases based on a trimmed alignment of 283 amino acids. Nodes exhibiting maximal bootstrap support and posterior probability (PP) are marked by black circles. Only bootstrap supports ≥50 and PP values ≥0.5 are shown. Clades of eukaryotic sequences are highlighted in yellow. Euglenozoan sequences analyzed in this study are shown on magenta background. Figure S9. A phylogenetic tree of D-lactate dehydrogenase sequences based on a trimmed alignment of 406 amino acids. Nodes exhibiting maximal bootstrap support and posterior probability (PP) are marked by black circles. Only bootstrap supports ≥50 and PP values ≥0.5 are shown. Clades of eukaryotic sequences are highlighted in yellow. Euglenozoan sequences analyzed in this study are shown on magenta background. Figure S10. A phylogenetic tree of inositol monophosphatase-like histidinol-phosphate phosphatases based on a trimmed alignment of 248 amino acids. Nodes exhibiting maximal bootstrap support and posterior probability (PP) are marked by black circles. Only bootstrap supports ≥50 and PP values ≥0.5 are shown. Clades of eukaryotic sequences are highlighted in yellow. Euglenozoan sequences analyzed in this study are shown on magenta background. Figure S11. A phylogenetic tree of histidinol-phosphate phosphatases belonging to the polymerase and histidinol-phosphate phosphatase protein family based on a trimmed alignment of 247 amino acids. Nodes exhibiting maximal bootstrap support and posterior probability (PP) are marked by black circles. Only bootstrap supports ≥50 and PP values ≥0.5 are shown. Clades of eukaryotic sequences are highlighted in yellow. Euglenozoan sequences analyzed in this study are shown on magenta background. [file 12915_2020_754_MOESM11_ESM.pdf]

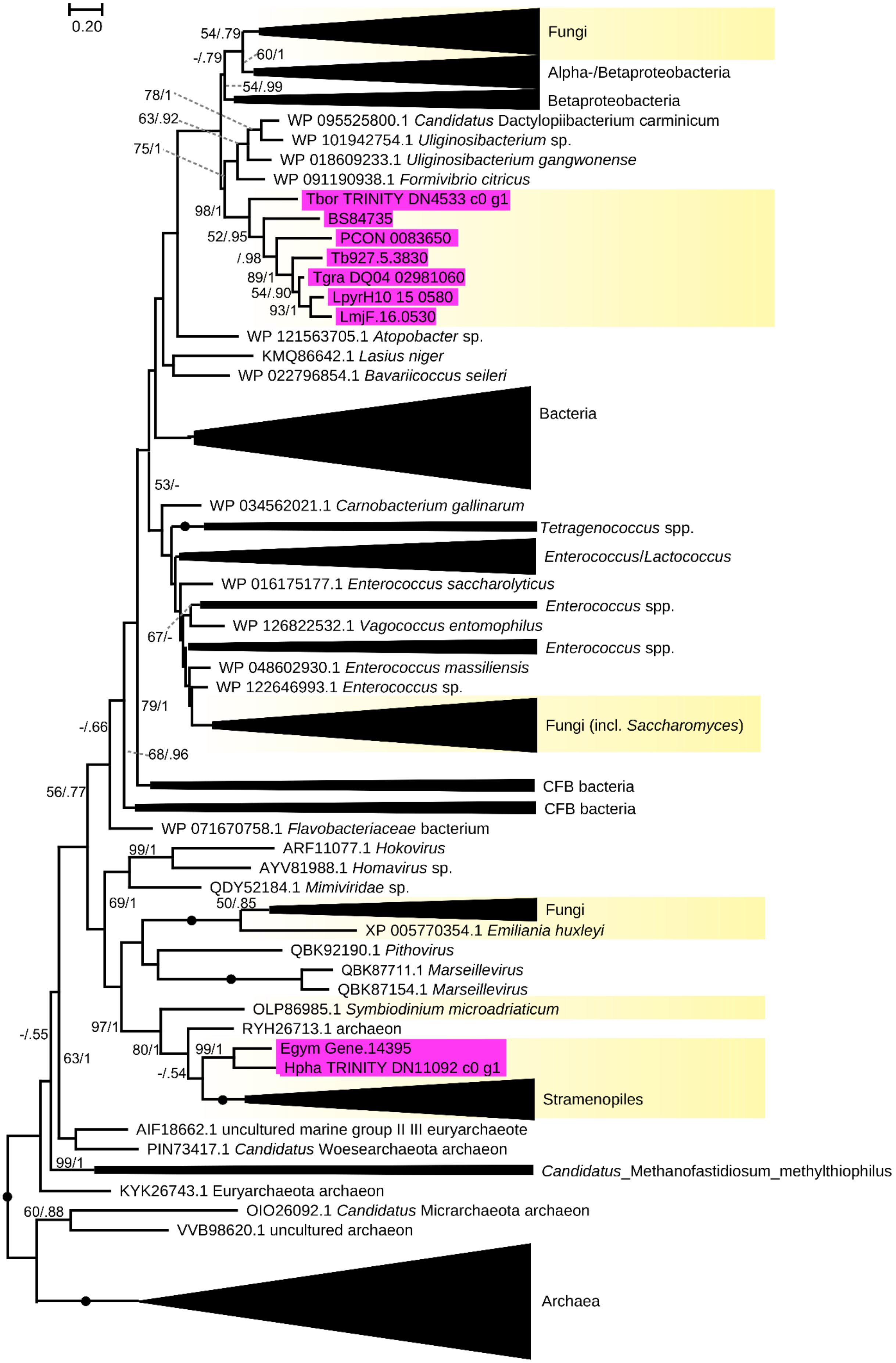
0.20
Fungi
Alpha-/Betaproteobacteria
Betaproteobacteria
54/.79
-/.79
60/1
54/.99
78/1
63/.92
75/1
WP 095525800.1 Candidatus Dactylopiibacterium carminicum
WP 101942754.1 Uliginosibacterium sp.
WP 018609233.1 Uliginosibacterium gangwonense
WP 091190938.1 Formivibrio citricus
98/1
52/.95
/.98
89/1
54/.90
93/1
Tbor TRINITY DN4533 c0 g1
BS84735
PCON 0083650
Tb927.5.3830
Tgra DQ04 02981060
LpyrH10 15 0580
LmjF.16.0530
WP 121563705.1 Atopobacter sp.
KMQ86642.1 Lasius niger
WP 022796854.1 Bavariicoccus seileri
Bacteria
53/-
WP 034562021.1 Carnobacterium gallinarum
Tetragenococcus spp.
Enterococcus/Lactococcus
WP 016175177.1 Enterococcus saccharolyticus
Enterococcus spp.
67/-
WP 126822532.1 Vagococcus entomophilus
Enterococcus spp.
WP 048602930.1 Enterococcus massiliensis
WP 122646993.1 Enterococcus sp.
79/1
Fungi (incl. Saccharomyces)
-/.66
68/.96
CFB bacteria
56/.77
CFB bacteria
WP 071670758.1 Flavobacteriaceae bacterium
99/1
ARF11077.1 Hokovirus
AYV81988.1 Homavirus sp.
QDY52184.1 Mimiviridae sp.
69/1
50/.85
Fungi
XP 005770354.1 Emiliania huxleyi
QBK92190.1 Pithovirus
QBK87711.1 Marseillevirus
QBK87154.1 Marseillevirus
OLP86985.1 Symbiodinium microadriaticum
-/.55
97/1
RYH26713.1 archaeon
80/1
99/1
Egym Gene.14395
63/1
-/.54
Hpha TRINITY DN11092 c0 g1
Stramenopiles
AIF18662.1 uncultured marine group II III euryarchaeote
PIN73417.1 Candidatus Woesearchaeota archaeon
99/1
Candidatus_Methanofastidiosum_methylthiophilus
KYK26743.1 Euryarchaeota archaeon
60/.88
OIO26092.1 Candidatus Micrarchaeota archaeon
VVB98620.1 uncultured archaeon
Archaea

Figure S8. A phylogenetic tree of fumarate-dependent dihydroorotate dehydrogenases based on a trimmed alignment of 283 amino acids. Nodes exhibiting maximal bootstrap support and posterior probability (PP) are marked by black circles. Only bootstrap supports ≥50 and PP values ≥0.5 are shown**.** Clades of eukaryotic sequences are highlighted in yellow. Euglenozoan sequences analyzed in this study are shown on magenta background.

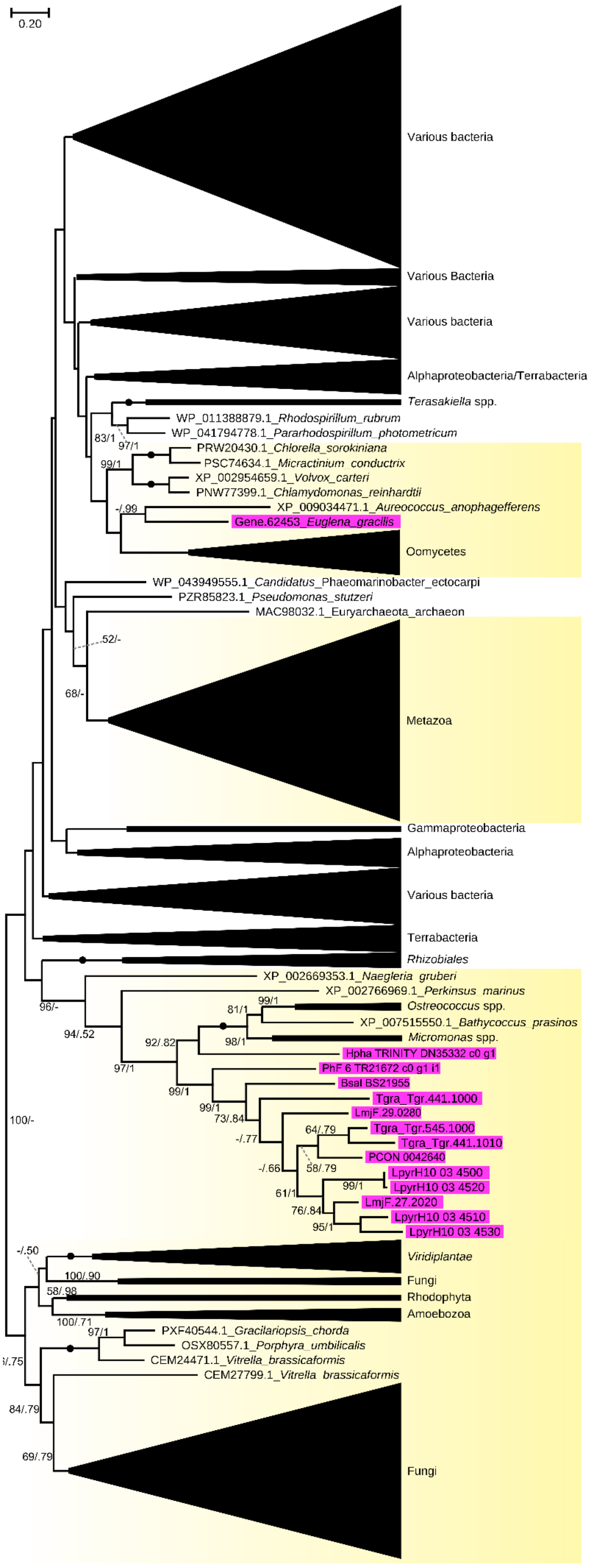
0.20
Various bacteria
Various Bacteria
Various bacteria
Alphaproteobacteria/Terrabacteria
Terasakiella spp.
WP_011388879.1_Rhodospirillum_rubrum
WP_041794778.1_Pararhodospirillum_photometricum
83/1
97/1
PRW20430.1_Chlorella_sorokiniana
PSC74634.1_Micractinium_conductrix
99/1
XP_002954659.1_Volvox_carteri
PNW77399.1_Chlamydomonas_reinhardtii
-/.99
XP_009034471.1_Aureococcus_anophagefferens
Gene.62453_Euglena_gracilis
Oomycetes
WP_043949555.1_Candidatus_Phaeomarinobacter_ectocarpi
PZR85823.1_Pseudomonas_stutzeri
MAC98032.1_Euryarchaeota_archaeon
.52/-
68/-
Metazoa
Gammaproteobacteria
Alphaproteobacteria
Various bacteria
Terrabacteria
Rhizobiales
XP_002669353.1_Naegleria_gruberi
XP_002766969.1_Perkinsus_marinus
96/-
99/1
81/1
Ostreococcus spp.
94/.52
XP_007515550.1_Bathycoccus_prasinos
98/1
Micromonas spp.
92/.82
Hpha TRINITY DN35332 c0 g1
97/1
PhF 6 TR21672 c0 g1 i1
99/1
Bsal BS21955
99/1
Tgra_Tgr.441.1000
73/.84
LmjF.29.0280
100/-
-/.77
64/.79
Tgra_Tgr.545.1000
Tgra_Tgr.441.1010
PCON 0042640
-/.66
58/.79
LpyrH10 03 4500
99/1
LpyrH10 03 4520
61/1
LmjF.27.2020
76/.84
LpyrH10 03 4510
95/1
LpyrH10 03 4530
-/.50
Viridiplantae
100/.90
Fungi
58/.98
Rhodophyta
100/.71
Amoebozoa
97/1
PXF40544.1_Gracilariopsis_chorda
OSX80557.1_Porphyra_umbilicalis
CEM24471.1_Vitrella_brassicaformis
CEM27799.1_Vitrella_brassicaformis
84/.79
69/.79
Fungi

Figure S9. A phylogenetic tree of D-lactate dehydrogenase sequences based on a trimmed alignment of 406 amino acids. Nodes exhibiting maximal bootstrap support and posterior probability (PP) are marked by black circles. Only bootstrap supports ≥50 and PP values ≥0.5 are shown**.** Clades of eukaryotic sequences are highlighted in yellow. Euglenozoan sequences analyzed in this study are shown on magenta background.

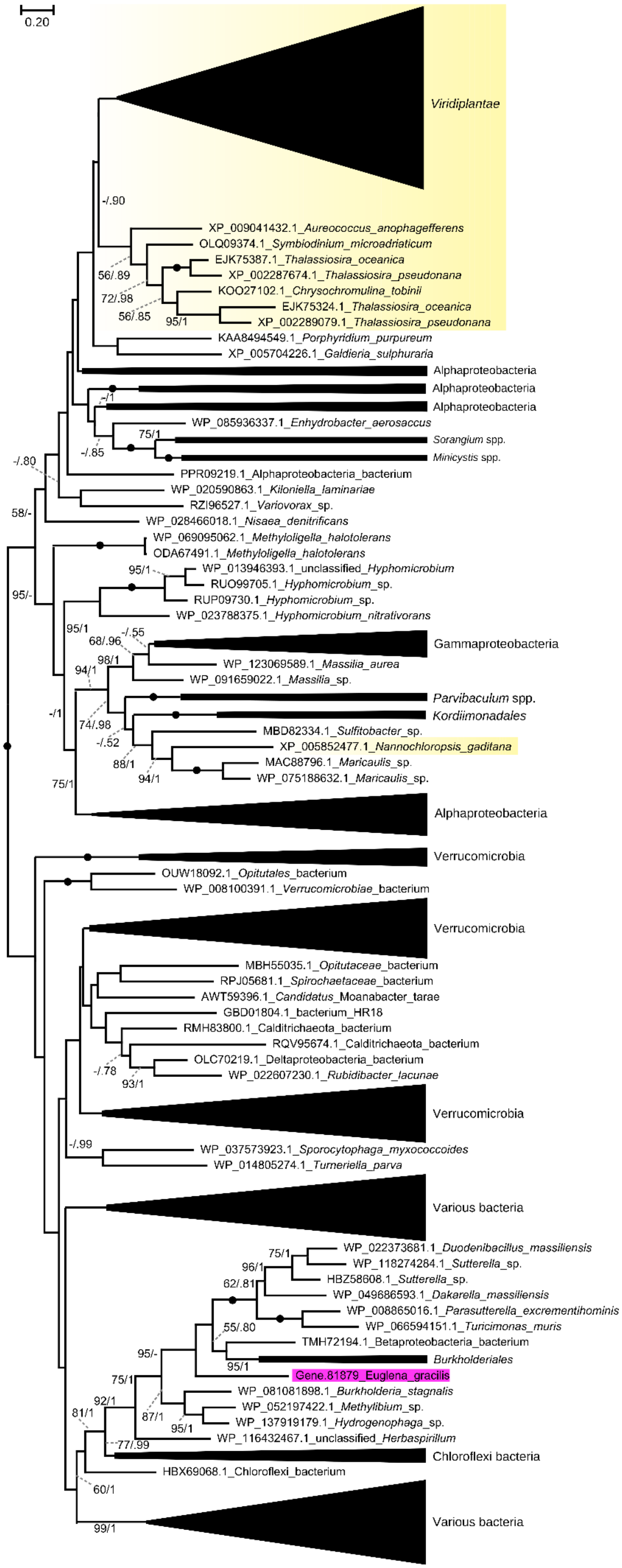
0.20
Viridiplantae
-/.90
XP_009041432.1_Aureococcus_anophagefferens
OLQ09374.1_Symbiodinium_microadriaticum
EJK75387.1_Thalassiosira_oceanica
XP_002287674.1_Thalassiosira_pseudonana
KOO27102.1_Chrysochromulina_tobinii
EJK75324.1_Thalassiosira_oceanica
XP_002289079.1_Thalassiosira_pseudonana
56/.89
72/.98
56/.85
95/1
KAA8494549.1_Porphyridium_purpureum
XP_005704226.1_Galdieria_sulphuraria
Alphaproteobacteria
Alphaproteobacteria
-/1
Alphaproteobacteria
WP_085936337.1_Enhydrobacter_aerosaccus
75/1
Sorangium spp.
-/.85
Minicystis spp.
-/.80
PPR09219.1_Alphaproteobacteria_bacterium
WP_020590863.1_Kiloniella_laminariae
RZI96527.1_Variovorax_sp.
58/-
WP_028466018.1_Nisaea_denitrificans
WP_069095062.1_Methyloligella_halotolerans
ODA67491.1_Methyloligella_halotolerans
95/1
WP_013946393.1_unclassified_Hyphomicrobium
RUO99705.1_Hyphomicrobium_sp.
95/-
RUP09730.1_Hyphomicrobium_sp.
WP_023788375.1_Hyphomicrobium_nitrativorans
95/1
-/.55
68/.96
Gammaproteobacteria
98/1
WP_123069589.1_Massilia_aurea
94/1
WP_091659022.1_Massilia_sp.
Parvibaculum spp.
-/1
Kordiimonadales
74/.98
MBD82334.1_Sulfitobacter_sp.
-/.52
XP_005852477.1_Nannochloropsis_gaditana
88/1
MAC88796.1_Maricaulis_sp.
94/1
WP_075188632.1_Maricaulis_sp.
75/1
Alphaproteobacteria
Verrucomicrobia
OUW18092.1_Opitutales_bacterium
WP_008100391.1_Verrucomicrobiae_bacterium
Verrucomicrobia
MBH55035.1_Opitutaceae_bacterium
RPJ05681.1_Spirochaetaceae_bacterium
AWT59396.1_Candidatus_Moanabacter_tarae
GBD01804.1_bacterium_HR18
RMH83800.1_Calditrichaeota_bacterium
RQV95674.1_Calditrichaeota_bacterium
OLC70219.1_Deltaproteobacteria_bacterium
-/.78
WP_022607230.1_Rubidibacter_lacunae
93/1
Verrucomicrobia
-/.99
WP_037573923.1_Sporocytophaga_myxococcoides
WP_014805274.1_Turneriella_parva
Various bacteria
75/1
WP_022373681.1_Duodenibacillus_massiliensis
96/1
WP_118274284.1_Sutterella_sp.
HBZ58608.1_Sutterella_sp.
62/.81
WP_049686593.1_Dakarella_massiliensis
WP_008865016.1_Parasutterella_excrementihominis
WP_066594151.1_Turicimonas_muris
55/.80
TMH72194.1_Betaproteobacteria_bacterium
95/-
Burkholderiales
95/1
Gene.81879_Euglena_gracilis
75/1
WP_081081898.1_Burkholderia_stagnalis
92/1
WP_052197422.1_Methylibium_sp.
81/1
87/1
WP_137919179.1_Hydrogenophaga_sp.
95/1
WP_116432467.1_unclassified_Herbaspirillum
77/.99
Chloroflexi bacteria
HBX69068.1_Chloroflexi_bacterium
60/1
Various bacteria
99/1

Figure S10. A phylogenetic tree of inositol monophosphatase-like histidinol-phosphate phosphatases based on a trimmed alignment of 248 amino acids. Nodes exhibiting maximal bootstrap support and posterior probability (PP) are marked by black circles. Only bootstrap supports ≥50 and PP values ≥0.5 are shown**.** Clades of eukaryotic sequences are highlighted in yellow. Euglenozoan sequences analyzed in this study are shown on magenta background.

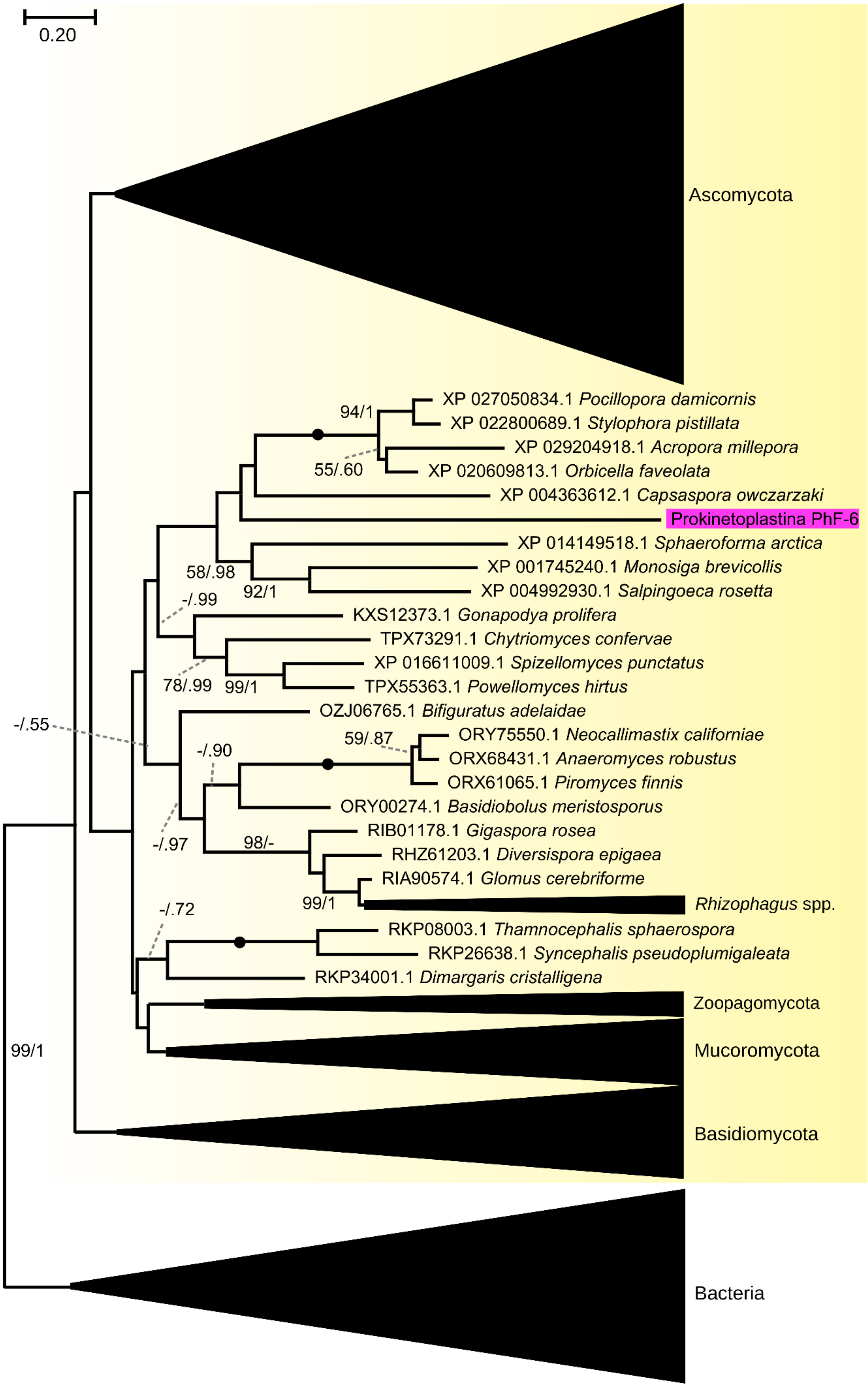
0.20
Ascomycota
94/1
XP 027050834.1 Pocillopora damicornis
XP 022800689.1 Stylophora pistillata
XP 029204918.1 Acropora millepora
55/.60
XP 020609813.1 Orbicella faveolata
XP 004363612.1 Capsaspora owczarzaki
Prokinetoplastina PhF-6
XP 014149518.1 Sphaeroforma arctica
58/.98
XP 001745240.1 Monosiga brevicollis
92/1
XP 004992930.1 Salpingoeca rosetta
-/.99
KXS12373.1 Gonapodya prolifera
TPX73291.1 Chytriomyces confervae
XP 016611009.1 Spizellomyces punctatus
78/.99
99/1
TPX55363.1 Powellomyces hirtus
OZJ06765.1 Bifiguratus adelaidae
-/.55
59/.87
ORY75550.1 Neocallimastix californiae
-/.90
ORX68431.1 Anaeromyces robustus
ORX61065.1 Piromyces finnis
ORY00274.1 Basidiobolus meristosporus
RIB01178.1 Gigaspora rosea
-/.97
98/-
RHZ61203.1 Diversispora epigaea
RIA90574.1 Glomus cerebriforme
99/1
Rhizophagus spp.
-/.72
RKP08003.1 Thamnocephalis sphaerospora
RKP26638.1 Syncephalis pseudoplumigaleata
RKP34001.1 Dimargaris cristalligena
Zoopagomycota
99/1
Mucoromycota
Basidiomycota
Bacteria

Figure S11. A phylogenetic tree of histidinol-phosphate phosphatases belonging to the polymerase and histidinol-phosphate phosphatase protein family based on a trimmed alignment of 247 amino acids. Nodes exhibiting maximal bootstrap support and posterior probability (PP) are marked by black circles. Only bootstrap supports ≥50 and PP values ≥0.5 are shown**.** Clades of eukaryotic sequences are highlighted in yellow. Euglenozoan sequences analyzed in this study are shown on magenta background.
